# BrAD-seq: Breath Adapter Directional sequencing: a streamlined, ultra-simple and fast library preparation protocol for strand specific mRNA library construction

**DOI:** 10.3389/fpls.2015.00366

**Published:** 2015-05-22

**Authors:** Brad T. Townsley, Michael F. Covington, Yasunori Ichihashi, Kristina Zumstein, Neelima R. Sinha

**Affiliations:** Department of Plant Biology, University of California, DavisDavis, CA, USA

**Keywords:** strand-specific sequencing, NGS, Illumina, RNA-seq libraries, Bioinformatics, BrAD-seq

## Abstract

Next Generation Sequencing (NGS) is driving rapid advancement in biological understanding and RNA-sequencing (RNA-seq) has become an indispensable tool for biology and medicine. There is a growing need for access to these technologies although preparation of NGS libraries remains a bottleneck to wider adoption. Here we report a novel method for the production of strand specific RNA-seq libraries utilizing the terminal breathing of double-stranded cDNA to capture and incorporate a sequencing adapter. Breath Adapter Directional sequencing (BrAD-seq) reduces sample handling and requires far fewer enzymatic steps than most available methods to produce high quality strand-specific RNA-seq libraries. The method we present is optimized for 3-prime Digital Gene Expression (DGE) libraries and can easily extend to full transcript coverage shotgun (SHO) type strand-specific libraries and is modularized to accommodate a diversity of RNA and DNA input materials. BrAD-seq offers a highly streamlined and inexpensive option for RNA-seq libraries.

## Introduction

Next Generation Sequencing (NGS) technologies have rapidly become foundational tools of genomics research (Koboldt et al., 2013). In particular, RNA-sequencing (RNA-seq) has transformed gene expression analyses and promoted the study of non-model organisms at an unprecedented level of detail with the ability to generate transcriptome assemblies for virtually any species (Sémon, 2014). On the most commonly used Illumina platform the ability to sequence a large number of biological samples requires the creation of libraries from nucleic acid samples with specified sequence “adapters” at the termini of the molecules. There are a variety of methods available to generate adapter-added libraries from nucleic acid samples from a variety of source materials, however the process still remains technically challenging, laborious, and expensive, thereby limiting widespread access to the technology.

Here we present a novel and efficient method for constructing strand specific RNA-seq libraries in a simple, rapid, and inexpensive modular format. The method is optimized to create strand specific 3-prime Digital Gene Expression (DGE—providing readout from the 3′ end of the mRNA) and can be adapted for strand-specific non-DGE shotgun type (SHO) and more conventional non-strand specific (CNV) RNA-seq libraries, in addition to utilizing a variety of DNA source materials. 3-prime DGE libraries are often preferred for gene expression studies because a single mRNA yields approximately 1 sequence read reducing potential sources of bias.

Strand specific RNA-seq requires the directional addition of unique 5-prime and 3-prime adapter sequences during preparation of the cDNA libraries. This is accomplished in a number of ways among the various NGS library preparation protocols. These include, the ligation of a known sequence to the 5-prime portion of mRNA molecules prior to cDNA synthesis (Lister et al., 2008), removal of the template RNA strand followed by randomly primed 2nd strand synthesis (Armour et al., 2009), labeling of first or second strand cDNA molecules with dUTP for enzymatic degradation prior to enrichment (Parkhomchuk et al., 2009) and the use of terminal transferases to add defined nucleotides to the cDNA molecules (Zhu et al., 2001; Tang et al., 2010), with each method having advantages and shortcomings (Levin et al., 2010). Our method for directional NGS library construction considerably simplifies and accelerates the library construction process. Only around 10 milligrams of cytoplasmically dense plant tissue such as Shoot Apical Meristem (SAM) or leaf primordia (slightly larger amounts for mature tissue), are required for RNA-seq library production, and an individual worker can readily complete the procedure starting from tissue in a single day.

We utilize an aspect of nucleic acid chemistry that has not been exploited in available methods to generate strand specific libraries. Double stranded nucleic acids undergo a phenomenon called “breathing” where the individual strands will momentarily separate to expose the bases (von Hippel et al., 2013). This process happens at a higher rate at the ends of double stranded nucleic acids (von Hippel et al., 2013). We exploit this transient terminal breathing to incorporate an adapter oligonucleotide that includes the Illumina TruSeq PE1 sequence specifically at the 5-prime terminus of the RNA-cDNA duplex. Breath capture allows for streamlined strand-specific library protocols not requiring prior second strand synthesis or removal of template RNA, allowing construction of either 3-prime DGE or shotgun (SHO) type strand specific libraries.

From these basic strand specific modules we further developed additional compatible modules to accommodate a variety of nucleic acid species as input materials—single-stranded RNA, double-stranded DNA, and single-stranded DNA. This provides a general purpose platform for creation of libraries for gene expression studies, genomic DNA libraries as well as from the products of amplification of minute samples such as DNA obtained in Chromatin Immunoprecipitation (ChIP) experiments and RNA from Laser Capture Microdissected (LCM) tissue samples. The use of common modules in this platform minimizes the number of individual reagents required to generate any number of library types, as well as standardizes the handling and manipulation steps, reducing the learning curve and minimizing the potential for human error.

## Materials and methods

**Figure 1:**
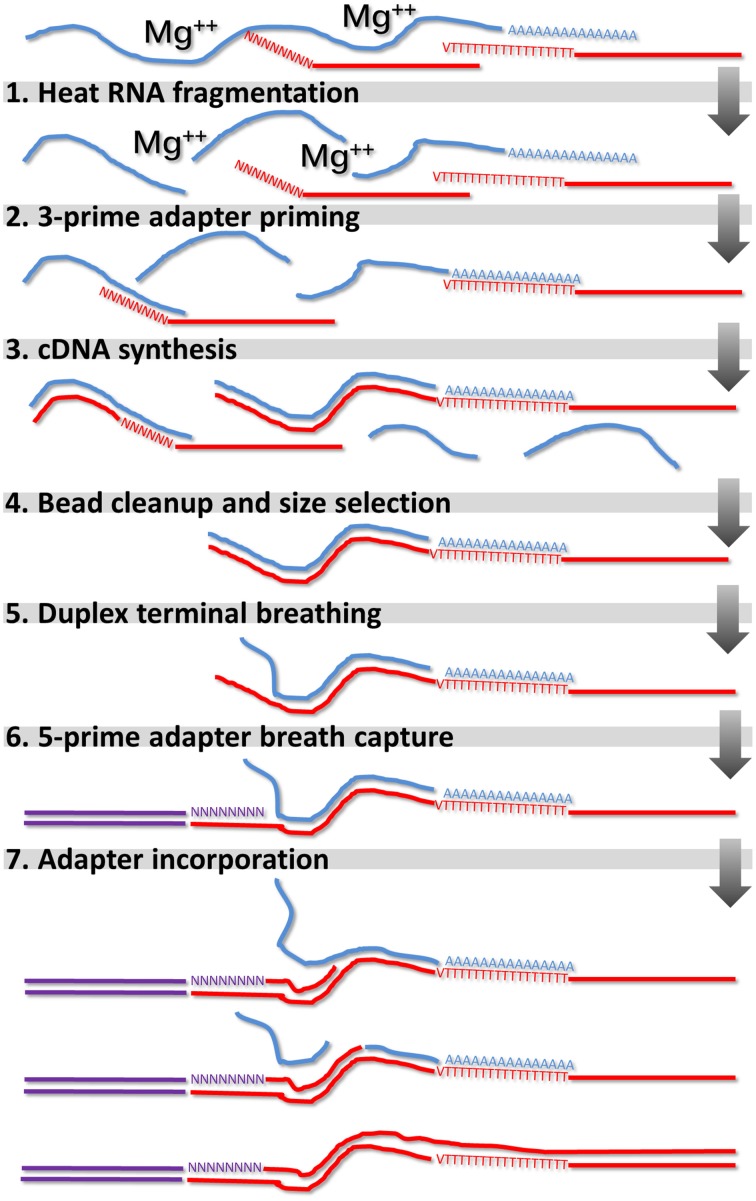
**Schematic diagram of strand-specific library synthesis mechanism**. mRNAs are fragmented by heat and magnesium **(1)** and primed for cDNA synthesis by an adapter-containing oligonucleotide **(2,3)**. Size selection and cleanup removes unincorperated oligonucleotides and small cDNA fragments **(4)**. Transient duplex breathing at the terminus of the RNA-cDNA hybrid **(5)** facilitates interaction with the single-stranded portion of the 5-prime capturing adapter **(6)** and *E. coli* DNA Polymerase I catalyses its incorporation into a complete library molecule **(7)**.

A schematic diagram of the reaction steps for strand-specific library synthesis is shown in Figure 1. Brief protocol for non-strand specific “conventional” (CNV) RNA-seq libraries can be found in Supplementary Methods 1. Detailed directions for strand specific DGE RNA-seq as well as strand specific SHO RNA-seq and non-strand CNV RNA-seq and DNA-seq protocol variants can be found in Supplementary Methods 2. All oligonucleotides used in this study were ordered from Life Technologies (Thermo Fisher Scientific) at 50 nanomole scale, desalted with no additional purification.

### Plant material

Tomato seeds (*S. lycopersicum* cv M82: LA3475) were provided by the Tomato Genetics Resource Center, University of California, Davis. After sterilization (50% bleach for 1 min followed by rinse with water), seeds were placed onto water-soaked paper towels in Phytatrays (Sigma) in the dark for 3 days at room temperature to allow germination. The germinated seeds within Phytatrays were placed into a growth chamber at 22°C with 70% relative humidity and a photoperiod of 16 h light/8 h dark for another 4 days. Seedlings were then transplanted into Sunshine Mix soil (Sun Gro). After growing in soil for 11 days, P5 leaf primordia (the leaf sample) and SAM (consisting of the SAM and 4 younger leaf primordia) were dissected carefully using razor blades and harvested into RNase-free tubes.

### mRNA isolation

Tissues were processed and lysed as described previously by Kumar et al. (2012) using zircon beads and Lysate Binding Buffer containing Sodium dodecyl sulfate in place of Lithium dodecyl sulfate. mRNA was isolated from 200 μl of lysate per sample. 1 μl of 12.5 μM of 5-prime biotinylated polyT oligonucleotide containing a 5-prime 20 nucleotide arbitrary spacer sequence followed by 20 thymidine nucleotides (5′-bio- ACAGGACATTCGTCGCTTCCTTTTTTTTTTTTTTTTTTTT-3′) was added to each lysate sample, mixed by pipetting several times and allowed to stand for 10 min. Following incubation, captured mRNAs were isolated from the lysate by the addition of 20 μl of LBB washed Streptavidin-coated magnetic beads (New England BioLabs, Cat. # S1420S). The bead-lysate mixture was mixed by pipetting and allowed to stand an additional 10 min. Samples were placed on a 96-well magnetic separator (Edge BioSystems, Cat. # 57624) and washed as previously described (Kumar et al., 2012) with the following modifications. (A) Wash volumes of WBA, WBB, and LSB were 300 μl each and buffers were chilled on ice prior to use. (B) mRNA elution was done into 16 μl of 10 mM TrisHCl pH 8 containing 1 mM β-mercaptoethanol.

### mRNA fragmentation, 3-prime adapter priming and cDNA synthesis

mRNA fragmentation was accomplished using magnesium ions (3 mM) at elevated temperature (Supplementary Figure 1) and was standardized at 90 s. Priming for the cDNA synthesis reaction was carried out in a single reaction mixture for Strand Specific-DGE, Strand Specific-SHO, and non-Strand Specific libraries were fragmented in a reaction containing 1.5 μl 5X RT buffer (Thermo scientific, Cat. # EP0441), 1 μl of priming adapter and 7.5 μl of the sample mRNA in a total reaction volume of 10 μl. Mixtures were spun down and incubated in a thermocycler. The following oligonucleotides and thermocycler programs were used for each library type. Additional details and comments can be found in Supplementary Methods 2.

**DGE**: 1 μl of 2 μM oligo L-3ILL-20TV.2 (5′-GTGACTGGAGTTCAGACGTGTGCTCTTCCGATCTTTTTTTTTTTTTTTTTTTTV-3′) (25°C 1 s, 94°C 1.5 min, 30°C 1 min, 20°C 4 min, 20°C hold).

**SHO**: 1 μl of 5 μM oligo L-3ILL-N8.2 (5′-GTGACTGGAGTTCAGACGTGTGCTCTTCCGATCTNNNNNNNN-3′) (25°C 1 s, 94°C 1.5 min, 4°C 5 min, 20°C hold).

### cDNA synthesis

cDNA was synthesized by addition of 5 μl of the following reaction mixture to the fragmented and primed mRNA: 1.5 μl 5X Thermo Scientific RT buffer (Thermo scientific, Cat. # EP0441), 1.5 μl 0.1 M Dithiothreitol (DTT), 1 μl H_2_O, 0.5 μl 25 mM dNTPs (Thermo Scientific, Cat. # R1121), 0.5 μl RevertAid RT enzyme (Thermo scientific, Cat. # EP0441) (total reaction volume 15 μl). The reaction mixture was set up at room temperature and placed in a thermocycler running the following program: (25°C 10 min, 42°C 50 min, 50°C 10 min, 70°C 10 min, 4°C hold). cDNA was cleaned and size-selected prior to “breath capture” or second strand synthesis by addition of 5 μl 50 mM EDTA pH 8.0 and 30 μl Agencourt AMPure XP beads (Beckman, Cat. # A63881) to each sample and mixed by pipetting. After 5 min, samples were placed on a magnetic tray, supernatant was removed, and pellets were washed twice with 300 μl 80% ethanol without pellet disruption. Residual ethanol was removed with 20-μl pipette tip and samples were allowed to air-dry until no visible traces of liquid were detectable.

### 5-prime duplex breath capture adapter addition (strand specific)

5-prime adapter addition was done by rehydrating the cDNA bound to bead-pellet with 4 μl 10 μM pre-annealed 5-prime double stranded adapter oligo at room temperature. Double stranded 5-prime adapter was prepared by making a stock solution containing 10 mM each of oligos 5pSense8n and 5pAnti (5pSense8n 5′-CCTACACGACGCTCTTCCGATCTNNNNNNNN-3′, 5pAnti 5′-AGATCGGAAGAGCGTCGTGTAGG-3′) in H_2_O, dispensing to 100 μl volumes in strip tubes and annealing them in a thermocycler running the following program: [94 C, 1 min (94 C, 10 s) × 60 cycles -1 C/cycle, 20 C 1 min, 4 C hold]. Subsequently, 6 μl of the following reaction mixture was added, mixed by pipetting to fully re-suspend the pellet and incubated at room temperature for 15 min: 3.5 μl H_2_O, 1 μl 10X Thermo Pol I reaction buffer (Thermo Scientific, Cat. # EP0041), 1 μl 250 mM MgCl_2_ (made fresh and stored at −20 C), 0.25 μl 25 mM dNTPs (Thermo Scientific, Cat. # R1121), 0.25 μl Thermo DNA Pol I (Thermo Scientific, Cat. # EP0041) (10 μl total reaction volume). The pre-enrichment libraries on beads were washed and size-selected using Agencourt AMPure XP beads present from the previous step by adding 10 μl 50 mM EDTA pH 8.0 and 30 μl ABR solution (15% PEG 8000, 2.5 M NaCl), mixed thoroughly by pipetting and allowed to stand for 5 min prior to placing on the magnetic tray. Supernatant was removed and pellets were washed twice with 300 μl 80% ethanol, without pellet disruption. Residual ethanol was removed with 20-μl pipette tip and samples were allowed to air-dry until no visible traces of liquid were detectable. Pellets were re-suspended in 22 μl 10mM Tris pH 8.0, allowed to stand 1 min and place on the magnetic tray. Supernatant was transferred without beads to fresh strip tubes and stored at −20°C prior to enrichment.

For Conventional library steps please see Supplementary Methods 1 or the detailed protocol in Supplementary methods 2.

### PCR enrichment and index sequence addition (strand-specific and non-strand-specific)

The enrichment step was done using full length oligonucleotides containing the full adapter sequence as well as short oligonucleotides complementary to the distal-most portion of the adapter arms to ensure predominantly full-length amplification products. PCR enrichment was carried out by combining 1 μl of the 2 μM uniquely-indexed ILL-INDEX oligonucleotide (ILL-INDEX 5′-CAAGCAGAAGACGGCATACGAGATxxxxxxxxGTGACTGGAGTTCAGACGTGTGCTCTTCCGAT-3′) (Supplementary Table 1: Oligonucleotide sequences) with 9 μl of the master mix: 4 μl 5X Phusion HF Buffer, 2.6 μl H_2_O, 1 μl 2 μM PE1 primer (PE1 5′-AATGATACGGCGACCACCGAGATCTACACTCTTTCCCTACACGACGCTCTTCCGATCT-3′), 1 μl 8 μM each S1 + S2 primers (S1 5′-AATGATACGGCGACCACCGA-3′, S2 5′-CAAGCAGAAGACGGCATACGA-3′), 0.2 μl 25mM dNTPs, 0.2 μl Phusion Polymerase (Thermo scientific, Cat. # F-530L), and 10 μl of pre-enrichment cDNA in a total reaction volume of 20 μl. Half of the PCR mix (10 μl) was placed in separate sample tubes stored at −20°C as backup for samples where more cycles of enrichment were needed. The remaining 10 μl were spun down and placed in a thermocycler using the program: [98 C 30 s, (98 C 10 s, 65 C 30 s, 72 C 30 s) 11 cycles, 72 C 5 min, 10 C hold]. Samples showing only very faint enrichment were re-amplified with 14 cycles of enrichment from the backup PCR samples. 2 μl of each library sample was run on a 1% agarose gel, with 1 μl of O'GeneRuler 100 bp DNA ladder (Thermo Scientific, Cat. # SM1143) for size and quantity reference, at 100 volts for 20 min. The remaining 8 μl of enriched library sample was cleaned and size selected using 12 μl of fresh Agencourt AMPure XP beads and washing twice with 80% ethanol as in previous wash steps. The libraries were eluted from the pellet with 10 μl 10 mM Tris pH 8.0, quantified, and pooled as previously described (Kumar et al., 2012). 50 bp single end sequencing was carried out at the Vincent J. Coates Genomic sequencing Facility at UC Berkeley.

### Bioinformatics

Bioinformatics and statistical analysis was carried out using the iPlant Atmosphere cloud service (Goff et al., 2011). Reads were trimmed to 42 bp and quality filtered using FASTX-Toolkit (http://hannonlab.cshl.edu/fastx_toolkit/) and scripts developed by Comai lab, UC Davis (http://comailab.genomecenter.ucdavis.edu). Reads were mapped using Bowtie (Langmead et al., 2009) with the parameters specified in Supplementary Table 2. Read quality analysis was performed using FASTQC (http://www.bioinformatics.bbsrc.ac.uk/projects/fastqc/). The code that was used to perform each of the bioinformatic steps is available at https://github.com/plant-plasticity/townsley-fips-2015 and FASTQ files for RNA-seq data used in this study can be downloaded from Dryad data repository (http://datadryad.org/resource/doi:10.5061/dryad.9mq14).

## Results and discussion

To evaluate our strand-specific library preparation method, we prepared Shoot Apical Meristem (SAM) and leaf primordium (Leaf) samples using the new BrAD-seq DGE method and our previously-developed HTR method for a pairwise comparative analysis. In this protocol we add sample-identifying index sequences to the library molecules during the enrichment stage (Meyer and Kircher, 2010).

### Library enrichment

Although as a matter of procedure we don't typically quantify mRNA concentration prior to library synthesis to maintain higher throughput, when beginning experiments with unfamiliar materials it can be of utility to have some idea how many enrichment cycles would be reasonable to try. To ascertain the relationship between the input mRNA concentration and the number of enrichment cycles chosen, 22 mRNA samples which were used for DGE library synthesis were quantified on a Bioanalyzer using the RNA 6000 Pico kit (Agilent Technologies). This information was correlated with the number of cycles used for enrichment of each library sample and the concentration of washed libraries (Supplementary Figure 2). The relationship suggests that below about 10 ng/μl of mRNA it may be worthwhile to start with about 14 enrichment cycles at the first attempt, although individual preferences in interpretation of gel images and targeted final concentrations for pooling of samples will ultimately be important factors in deciding on the ideal number of enrichment cycles.

### Read quality

To avoid inclusion of sequence originating from the 5-prime adapter capture strand, the first 8 bases of DGE libraries was trimmed prior to analysis. For HTR libraries the percentage of reads mapping was also found to be higher (77.8 vs. 74.1%) when the first 8 bases were trimmed, so for all analyses trimmed FASTQ files were generated for samples prior to the quality filtering step. The mapping rate improves in trimmed HTR libraries because during cDNA synthesis random primers anneal with mismatches, incorperating non-native sequence into cDNA molecules.

**Figure 2:**
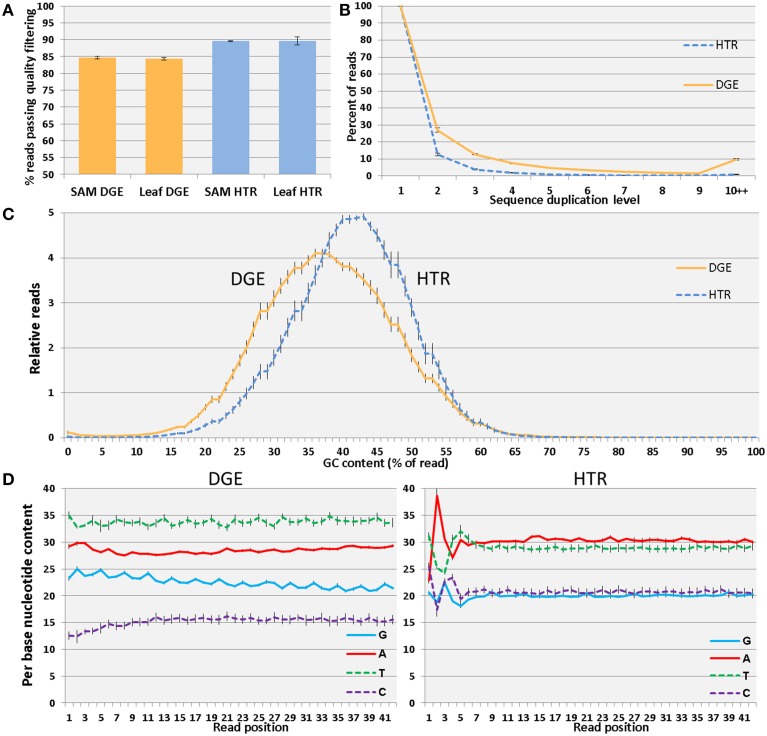
**Analysis of library quality and characteristics**. Percentage of reads passing all quality filtering steps **(A)**. Sequence duplication levels for DGE and HTR **(B)**. GC content of reads in DGE and HTR **(C)**. The average GC content is lower and the distribution broader in DGE than HTR. The composition of individual nucleotides differs between the strand-specific DGE and non-strand-specific HTR libraries **(D)**. Sequence bias is more evident in the HTR libraries in the first several positions of the trimmed quality-filtered reads. Error bars reflect standard deviation among samples separated by tissue and method **(A)** or by method **(B–D)**.

The overall quality scores for the raw DGE libraries was lower than HTR (Supplementary Figure 3) due to the inclusion of cDNA inserts containing polyA tracts. These low complexity sequences cannot be mapped to reference sequences and they are largely removed prior to mapping by quality filtering (Figure 2A and Supplementary Figure 3).

Since a population of strand-specific cDNA molecules highly enriched at the 3-prime of mRNA transcripts should be comprised of a smaller number of unique sequences for each transcript, identical reads from independent cDNA molecules are expected at a higher level than in non-strand-specific and non-DGE libraries. We do indeed observe higher sequence duplication for DGE and strand specific library types than HTR (Figure 2B). Non-DGE strand specific libraries have fuller transcript length coverage and show lower sequence duplication than DGE libraries resulting from higher sequence complexity (Supplementary Figure 4). Strand specific tomato SHO libraries made from similarly staged developing tomato leaves and Arabidopsis strand specific libraries(Hsu et al., 2013) downloaded from the Gene Expression Omnibus (Acession: GSE38879) made using a deoxy-Uracil (dU) marked strand specific method(Wang et al., 2011) were also assessed and possess similar rates of duplication to one another (Supplementary Figure 4). To remove differences in sequencing depth between samples as a factor in read duplication counts a random subsample of 1 million reads was used from each FASTQ file for duplication analysis.

Additionally, in 3-prime DGE libraries not all poly-A runs are removed by quality filtering. Homonucleotide “A” repeats make up the predominant duplicated sequences in DGE libraries, comprising ~0.3% of quality filtered reads. After quality-filtering, GC content and per base sequence content differ between DGE and HTR (Figure 2C) with lower GC content in strand-specific DGE library reads. Wheras individual base compositions in non-strand-specific libraries (e.g., HTR libraries) should contain roughly equal amounts of G to C and A to T nucleotides, G/C and A/T ratios are unequal for the coding strand of mRNAs. The proportions of each nucleotide in the sense strand of annotated tomato coding sequences is 22.1% G, 18.5% C, 29.9% A, 29.4% T. This closely matches the observed proportions in the DGE sequences: 22.5% G, 15.2% C, 28.5% A, 33.8% T (Figure 2D). Quality scores, sequence content and GC distribution show similar performance between SHO and dU library methods (Supplementary Figure 5).

### Adapter and rRNA contamination

**Figure 3:**
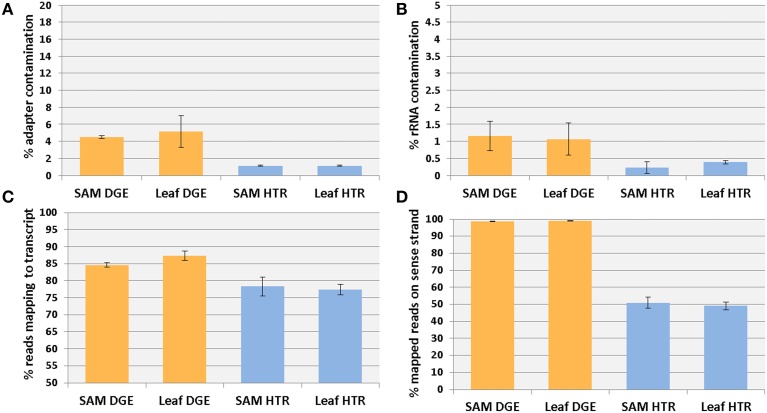
**Read mapping and strand specifity**. Fraction of reads coming from adapter **(A)** and ribosomal RNA **(B)** contamination. Reads mapping to either strand of ITAGcds + 500 reference **(C)**. Coding sequence mapped reads belonging to plus strand **(D)**.

Adapter contamination was higher in DGE libraries than in HTR (Figure 3A) consisting of ~5% of reads in DGE compared with ~1% of reads in HTR. This may be due to the use of higher PEG concentrations in the the bead washing step in the DGE protocol. This could increase bead binding of small products. Approximately 1% of reads from DGE libraries could be attributed to ribosomal contamination compared with 0.22 to 0.39% in HTR libraries (Figure 3B) and approximately 3% in tomato libraries made with a commercial Illumina kit (Kumar et al., 2012). Increased rRNA in DGE compared to HTR is lilely due to single step mRNA isolation compared to two stage mRNA re-isolation in the HTR process.

### Read mapping

To reliably compare DGE and HTR libraries we created a set of reference sequences consisting of the annotated tomato coding sequence plus an additional downstream portion corresponding to the genome sequence 3-prime to the stop codon. Plant 3-prime untranslated regions (3′-UTRs) are variable in length and average around 200 bp (Mignone et al., 2002) but many 3′-UTRs are not annotated. For the purpose of this study 500 bp of downstream genomic sequence was chosen to encompass most 3′-UTR sequences and appended to the annotated ITAG2.4 coding sequences (ITAGcds + 500). An additional mapping reference was generated specifically for DGE libraries consisting of the 3-prime 500 bp of the coding sequence plus an additional 500 bp representing the 3′-UTR (ITAG500 + 500) to minimize the effect of mis-priming of the 3-prime polyT containing adapter onto any A-rich regions within coding sequences.

The proportion of reads mapping one or more times to the plus and minus strands of the ITAGcds + 500 reference is higher in DGE (85–87%) than HTR (77–78%) (Figure 3C) demonstrating that a large majority of reads in both methods originate from mRNAs.

### DGE 3-prime selectivity

There is a strong selectivity of the DGE library protocol for the 3-prime portion of mRNA transcripts wheras reads derived from HTR are more evenly distributed across transcripts (Supplementary Figure 6). Although the ITAG500 + 500 reference sequences are, on average, 608 bp shorter than the ITAGcds + 500 reference sequences, more DGE reads map uniquely and strand-specifically to the ITAG500+500 reference (78–81%) than the HTR reads mapping uniquely to the ITAGcds + 500 reference (73–78%).

### Strand specifity

To evaluate strand-specificity of the DGE libraries, reads were mapped to tomato coding sequences only (Figure 3D) to exclude reads mapping to overlapping UTR regions. Approximately 99% of mapped reads in DGE libraries and 50% of mapped reads in HTR libraries localize to the sense strand, indicating a very high degree of strand-specificity for the DGE libraries. Directional information of the cDNA molecule is preserved because only the cDNA strand of the RNA-cDNA duplex can serve as a template for Pol I. We have successfully produced libraries using this method with *E. coli* Pol I, Klenow fragment, and Klenow exo- (Supplementary Figure 1C) indicating the exonuclease activity of Pol I is not required for the process to work efficiently.

**Figure 4:**
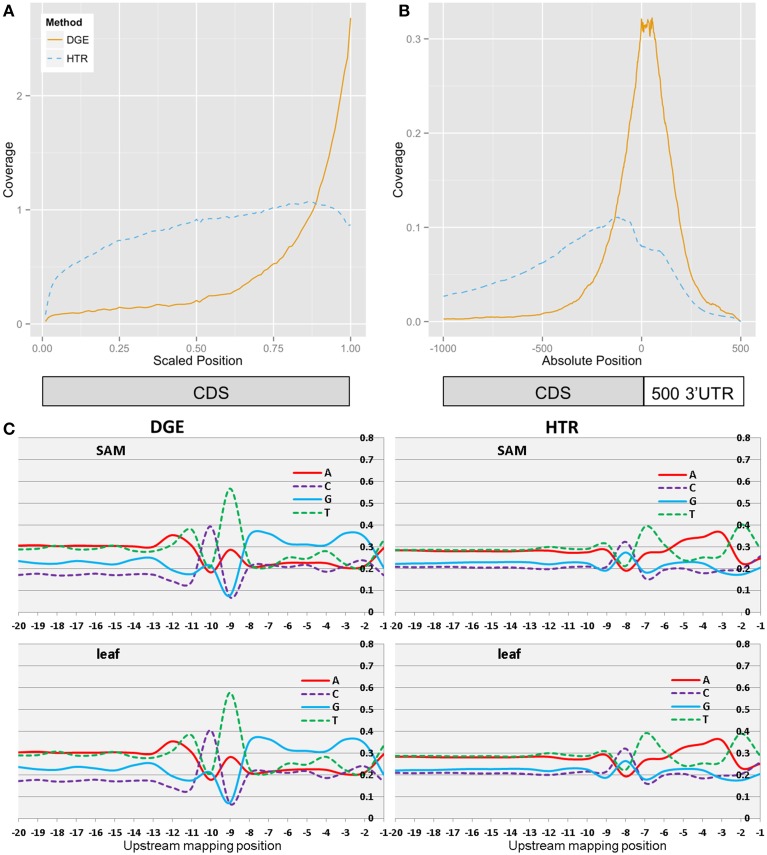
**Transcript coverage and cDNA sequence selection bias**. Localization of DGE and HTR reads within the mapping reference **(A)**, DGE reads mapped to 1.5 KB window localize near the annotated stop codon **(B)**. Base frequencies for transcript nucleotides upstream of mapped reads **(C)**.

A large majority of uniquely mapped reads (95%) in the DGE libraries map to a region ±500 bp of the annotated stop codons of ITAGcds + 500 reference (Table 1), whereas HTR libraries show a more even distribution across the transcript (Figure 4A). The DGE reads localize almost entirely to the 3-prime region of the transcript including downstream of the annotated stop codon, suggesting that only this interval is necessary for mapping DGE reads. HTR reads by comparison show a more even distribution but still bias toward sequence at the 3-prime of the transcript. Since not all coding sequences are 1 kb or longer the read locations were also scaled to the portion of the coding sequence (Figure 4B). HTR libraries still show a slight bias for sequences near the 3-prime end of the CDS. SHO libraries show similar transcript coverage to HTR although SHO coverage shows somewhat higher 5-prime transcript representation (Supplementary Figure 7).

**Table 1:** **DGE read mapping location in ITAGcds + 500 reference with respect to the stop codon**.

**Fraction of mapped reads%**	**Region of reference sequence**
>50	−60 to +120
>75	−150 to +200
>85	−250 to +250
>95	−500 to +500

To ascertain the degree of sequence selection bias introduced by the adapter capture process, 20 nucleotides upstream of the first mapped nucleotide for each read was extracted from the FASTA mapping reference for base composition (Figure 4C) and information content (Supplementary Figure 8). Positions −8 through −1 correspond to the cDNA region annealed to the 8 bp single stranded portion of the adapter responsible for breath capture of the DNA-RNA duplex. Positions −20 through −9 correspond to the “shielded” double stranded portion of the adapter containing the Illumina TruSeq PE1 sequence. Despite the presence of the shielding oligonucleotide, the positions approaching the −9 map location corresponding to the last few bases of the adapter show some sequence bias near the end of the double stranded region (Supplementary Figure 9). This suggests that duplex breathing of the adapter at the capturing end transiently exposes the first few internal bases, allowing for increased interaction with cDNA sequences with some complementarity. While the degree and range of this sequence selection bias is significantly improved over earlier versions of this protocol utilizing un-shielded single stranded adapters, it may still be further improved by converting the first base of the random 8mer into an extended double-stranded shield region. Retention of the template mRNA strand prevents access to the interior portions of the cDNA. This restricts the interactions of the adapter to the terminal portion of the cDNA, which provides control of library size through mRNA fragmentation and limits the effects of sequence specific secondary structures. Increasing Magnesium concentration in the breath capture reaction to 20 mM improves library yield (Supplementary Figure 1B) potentially through increased strength of base-pair interactions between the cDNA strand and the capturing adapter. The strand specificity of the DGE libraries also allows for unambiguous assignment of the transcript of origin for genes in which the terminator regions overlap (Supplementary Figure 8).

### Detection of gene expression

Reads were analyzed from equally-sized subsets of pre-quality-filtered reads (Table 2). The number of transcripts with mapped reads is reduced in both DGE and HTR libraries when excluding non-uniquely-mapped reads. The limited span of the transcript incorporated into DGE libraries, in combination with retaining only uniquely mapped reads and strand specificity may reduce the false detection of transcripts where genomic locations of transcripts overlap and where coding sequences are highly conserved.

**Table 2:** **Transcript detection for pre-quality-filtered subsets of 6.5 M reads each for DGE and HTR**.

			**Non-uniquely mapping**			**Uniquely mapping**					
			**Mapping to both strands**						**Maping to sense strand**		
			**ITAGcds+500**						**ITAGcds+500**					
**Combined sample**	**Initial reads**	**Passing QF**	**Mapped**	**Percent mapped**	**Transcripts detected**	**Reads mapping**	**Percent QF reads mapped**	**Transcripts with hits**	**Reads mapping**	**Percent QF reads mapped**	**Transcripts with hits**
DGE-SAM	6,500,000	5,255,791	4,449,163	85	23,348	4,252,370	81	21,618	4,113,253	78	20,922
DGE-Leaf	6,500,000	5,230,179	4,442,859	85	23,395	4,232,606	81	21,574	4,117,670	79	20,893
HTR-SAM	6,500,000	5,745,924	4,508,993	78	24,931	4,355,096	76	22,999			
HTR-Leaf	6,500,000	5,741,410	4,447,320	77	24,526	4,280,954	75	22,627			

*Non-uniquely mapping reads mapping to both strands of ITAGcds + 500 reference (Blue), uniquely mapping reads mapping to both strands of ITAGcds + 500 (Purple), and uniquely mapping reads mapping to sense strand of ITAG500 + 500 reference (Red)*.

**Figure 5:**
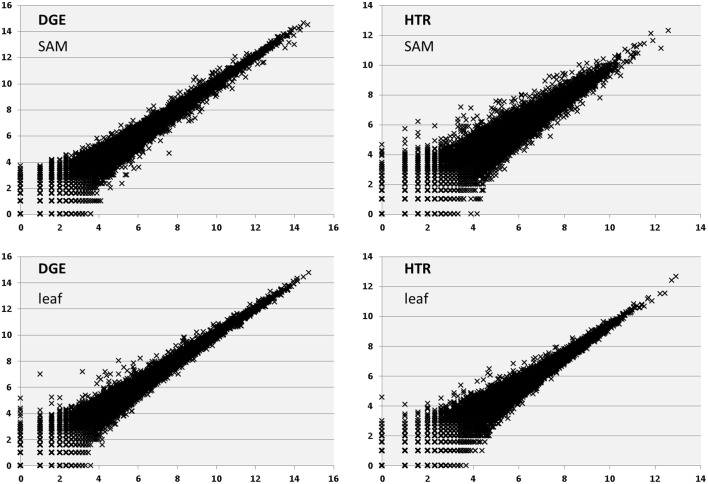
**Log2-transformed expression correlations for representative sample pairs for each sample DGE and HTR using a representative pair of samples for each**. Mean R-squared values for all DGE and HTR.

**Figure 6:**
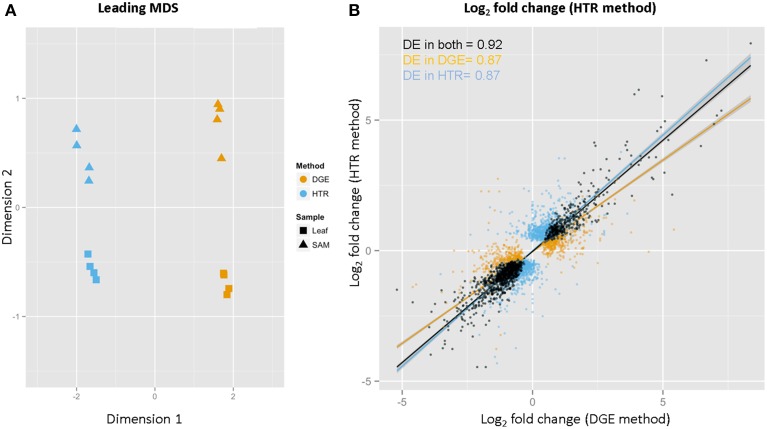
**Global comparisons of gene expression profiles between library construction methods**. Multi Dimensional Scaling (MDS) plot for DGE and HTR SAM and Leaf samples **(A)**. SAM vs. Leaf log_2_ fold change comparison between DGE and HTR **(B)**.

Correlation between replicates is higher for DGE than HTR samples (Figure 5 and Supplementary Table 3). R-squared values for all pairwise comparisons of Log2-transformed expression showed higher correlation between DGE (SAM 0.96, Leaf 0.95) replicates than HTR (SAM 0.91, Leaf 0.93). These values are also similar for DGE and *Arabidopsis* dU libraries (0.96) as well as between HTR and SHO (0.92). Variation between DGE and HTR experimental samples was also assessed using multidimensional scaling (MDS) (Figure 6A). Both DGE and HTR samples cluster by tissue type although distance between SAM and Leaf clusters is greater along dimension 2 for DGE libraries suggesting a high power of discrimination between tissues by gene expression. Differential gene expression calls between DGE and HTR show a high degree of overlap (Table 3). We found very strong correlation (*r*_s_ = 0.92) between the log_2_ fold-change of genes that are differentially regulated (FDR < 0.05) in SAM vs. leaf samples for both library preparation methods. The correlation remains very strong when considering genes differentially regulated for only the DGE method (*r*_s_ = 0.87; orange in Figure 6B) or only the HTR method (*r*_s_ = 0.87; blue in Figure 6B).

**Table 3:** **Differential gene expression calls for DGE and HTR library samples**.

**FDR 0.05**	**DGE Total**	**HTR total**	**DGE only**	**Both**	**HTR only**
Up (S vs. L)	2534	1386	1630	904	482
Down (S vs. L)	3014	1751	1615	1399	352
FDR 0.01					
Up (S vs. L)	1766	722	1251	515	207
Up (S vs. L)	2376	1128	1413	963	165

To compare within and across method differential expression results, we divided the samples into 10 groups of two replicates. The 10 sample groups were: 2 HTR leaf, 2 HTR SAM, 3 DGE leaf, and 3 DGE SAM. Within each library preparation method, we performed differential gene expression analysis for all combinations of leaf × SAM. This resulted in four comparisons for HTR and 9 for DGE. With these, we were able to calculate Spearman's Ranked Correlation Coefficient for all combinations of leaf-SAM differentially expressed genes within (45 for DGE and 6 for HTR) and between (36 for DGE vs. HTR) each library preparation method (Supplementary Figure 10). We found that although the fold-change of differentially regulated genes is less correlated when comparing between library preparation methods than within, both between- and within-method comparisons show very strongly correlation.

### Cost

We sought to minimize library prep cost and complexity by developing a protocol that uses mostly unmodified oligonucleotides and minimizes handling, steps, and reagents. The cost of isolating mRNA and making strand-specific libraries with this method is extraordinarily low, with magnetic bead, dNTP, and enzyme costs totaling $2.96/sample including mRNA isolation or $1.98 if making libraries from mRNA. Even allowing for the additional cost of consumables, chemical reagents and an extra 10% volume for reaction master mixes, this method provides a 20–40 fold cost reduction over available commercial strand-specific methods (e.g., NEBNext® Ultra™ Directional RNA Library Prep Kit for Illumina® 96 reactions Cat. # E7420L, SureSelect Strand Specific RNA-Seq Library Preparation kit for 96 samples reactions Cat. # G9691A).

### Protocol development

We had initially set out to modify a template switching protocol, but ended up making a discovery that would enable us to create arguably the cheapest and fastest RNA-seq protocol to date. Our original goal was to try to use adapter-encoded index sequences together with barcode sequences within the primary reads to achieve extremely dense multiplexing of samples. The 5-prime adapters were designed as single-stranded molecules with a partial Illumina PE1 sequence followed by a 9-base-pair sequence (a 6 base pair barcode and 3 terminal guanines) to facilitate base-pairing with non-templated cytosines added to the cDNA by MMLV polymerase. The addition of adapter sequence to the cDNA was done in a second reaction using *E. coli* Polymerase I following a size-selection bead cleanup to avoid “background cDNA” composed of adapter concatamers.

Our initial libraries showed a highly heterogeneous enrichment of identical pooled test mRNA dependent on the barcode sequence contained in the adapter (Supplementary Figure 11), with significant visible banding due to massive overrepresentation of specific amplicons which vary with the adapter barcode sequence. Following trimming of the first 9 nucleotides from the Illumina reads, mapping to tomato transcripts, and clustering of samples unexpectedly showed grouping based on barcode sequence and not on sample type (Supplementary Figure 12). Additionally, in the first attempt libraries only a small numbers of transcripts accounted for the majority of read counts.

Further investigation of these unexpected results showed that, while cDNA libraries that could be sequenced on the Illumina platform were produced, the priming mechanism did not utilize template switching as originally envisioned. Sequence analysis of the transcript reference sequences located 5-prime to the first mapped nucleotide of the trimmed reads showed an extreme bias in the sequenced tomato transcripts for nucleotides matching the barcode sequence and “G” repeats (Supplementary Figures 13–15) and further upstream sequences continued to include similarity to the PE1 sequence of the adapter. This indicated that base-pairing interactions between the terminal portion of the double-stranded cDNA and the barcode-containing portion of the adapter were selecting the transcripts that would be represented in the libraries.

Despite the rarity of any particular 9 base pair sequence in a given genome (one instance every 3.8e-06 bases), 74% of reads contained a perfect 9 base pair match to the barcode followed by 3 “G”s in the pre-trimmed portion of the read (Supplementary Figure 15). This showed that the dominant template for the sequencing reaction was the strand primed from 3-prime end of the adapter using the cDNA as a template. Consequently, the addition of non-templated “C”s by MMLV reverse transcriptase to the cDNA molecule likely blocked priming on the adapter oligonucleotide forcing the majority of sequenced molecules to originate from the second strand.

This suggested that there was a breathing effect in the double stranded template. We redesigned the 5-prime adapters to take advantage of this breath-capture effect and eliminate the sequence biases created by our early adapters. The portion of the adapter containing the Illumina PE1 sequence was shielded by annealing a complementary sequence oligonucleotide and the following 9 bases were replaced with variable length extensions of random mixed-base sequences, with extensions between 6 and 8 nucleotides outperforming shorter and longer variants. Adapter variants incorporating blocking groups at the 3-prime end of the random nucleotide extension performed extremely poorly indicating that priming from this strand was essential for library formation using this process.

## Conclusion

We have developed a rapid and inexpensive method for making strand-specific 3-prime DGE RNA-seq libraries from tissue in a multiplexed format. The entire process can be completed in a single working day. To our knowledge this is the first library construction process to utilize the terminal breathing of nucleic acid duplexes to selectively and directionally add adapter sequences. We have further developed the process to include modules allowing the creation of a variety of library types. We have also used the core DGE method on a number of species in addition to *S. lycopersicum* including *C. pentagona, S. pennellii, S. pimpinellifolium, S. neorickii*, and *N. tobacum*. To date we have successfully used our DGE protocol to study differential gene expression in a number of studies relating to development and abiotic stress with good results. We have added and adapted modules to this core protocol for our own purposes and we provide those modules as well so that others can also use this protocol as the basis for a universal RNA and DNA-seq library protocol family. In the hope of helping to democratize NGS sequencing technologies we offer an inexpensive and easily implemented protocol for the preparation of NGS libraries.

### Conflict of interest statement

The authors declare that the research was conducted in the absence of any commercial or financial relationships that could be construed as a potential conflict of interest.

## Supplementary material

Supplementary Figure 1 **RNA fragmentation by 3 mM magnesium at 94 degrees at increasing time intervals (A)**. Effect on library output of MgCl concentration in breath capture reaction using *E. coli* Polymerase I **(B)**. Breath capture reaction is successfully facilitated by *E. coli* polymerase I (2.5 U), Klenow fragment (1.25 U), and Klenow exo- (1.25 U) **(C)**. Lanes shown in C are 4, 2, and 2 technical replicates respectively. Breath capture reactions **(B,C)** were carried out at room temperature for 15 min.

Supplementary Figure 2 **RNA starting amounts vs library amplification, number cycles used, and concentration of washed libraries prior to pooling**.

Supplementary Figure 3 **Pre and post quality filtering PHRED scores for DGE and HTR libraries used in this study**.

Supplementary Figure 4 **Sequence duplication rates per million quality filtered reads**. High throughput (HTR) 23.12 % (Black dashed), DGE 66.15% (Orange solid), Shotgun (SHO) 53.63% (Yellow solid), deoxy-Uracil marked (dU) 48.28% (Blue solid).

Supplementary Figure 5 **FastQC analytics on filtered read information for additional strand specific library methods, Shotgun (SHO) (A,C,E), and deoxy-Uracil marked (dU) (B,D,F)**. Quality scores **(A,B)**, Base composition **(C,D)**, Percentage GC content **(E,F)**.

Supplementary Figure 6 **Genomic mapping location of uniquely mapped reads in DGE and HTR**. DGE reads show predominant localization to 3-prime of transcripts.

Supplementary Figure 7 **Transcript coverage trace for SHO libraries**.

Supplementary Figure 8 **Discrimination of read origin**. DGE reads can be positively assigned to their transcript of origin when transcripts overlap or are in close proximity by strand specificity of the reads.

Supplementary Figure 9 **Sequence logos displaying information content for 20 bases upstream of mapped reads**.

Supplementary Figure 10 **Pairwise comparisons of differential gene expression shows higher correlation within each method than between methods**.

Supplementary Figure 11 **Heterogeneous amplification from identical mRNA samples by single-stranded adapters containing barcode sequences near the 3-prime end**.

Supplementary Figure 12 **Hierarchical clustering of library samples made with single stranded barcode containing adapters shows grouping only by barcode sequence**.

Supplementary Figure 13 **Overrepresentation of reads mapping to positions containing Guanine repeats**.

Supplementary Figure 14 **Highly uneven distribution of mapping locations in libraries made with prototype adapters**.

Supplementary Figure 15 **Sequence information content for reads upstream of the first mapping nucleotide for the trimmed reads**.
