# Supplementary figures and images for: BrAD-seq: Breath Adapter Directional sequencing: a streamlined, ultra-simple and fast library preparation protocol for strand specific mRNA library construction
Source: Front Plant Sci. 2015 May 22;6:366. doi: 10.3389/fpls.2015.00366 (PMC4441129; doi:10.3389/fpls.2015.00366)

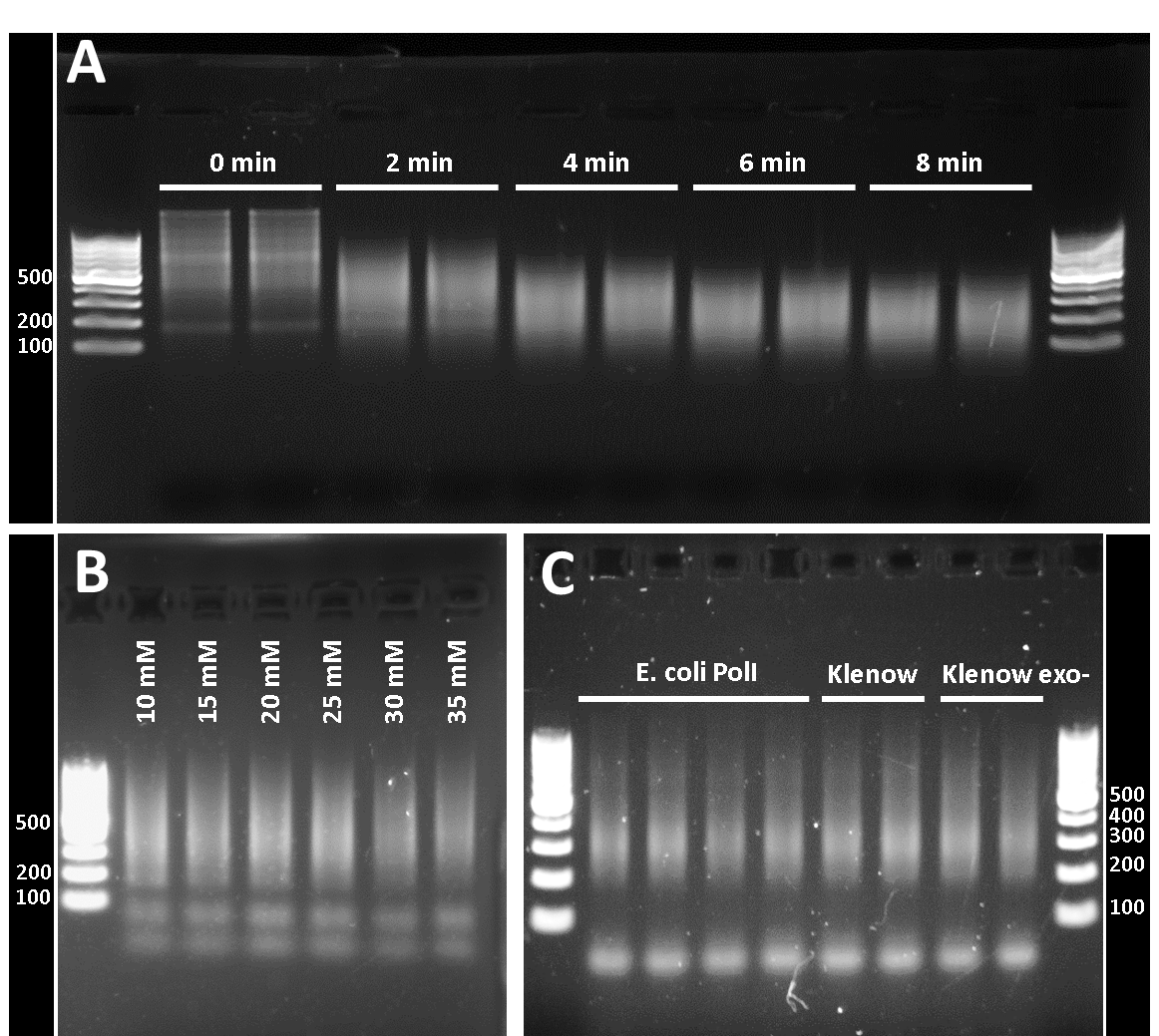

Supplement: Supplementary Figure 1 — RNA fragmentation by 3 mM magnesium at 94 degrees at increasing time intervals (A). Effect on library output of MgCl concentration in breath capture reaction using E. coli Polymerase I (B). Breath capture reaction is successfully facilitated by E. coli polymerase I (2.5 U), Klenow fragment (1.25 U), and Klenow exo- (1.25 U) (C). Lanes shown in C are 4, 2, and 2 technical replicates respectively. Breath capture reactions (B,C) were carried out at room temperature for 15 min. [file Image1.TIF]

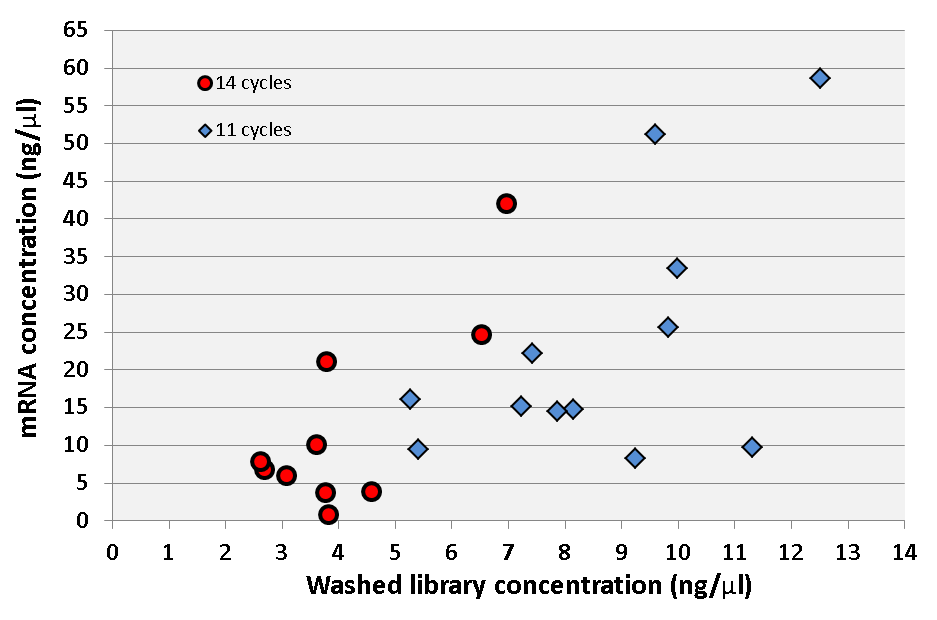

Supplement: Supplementary Figure 2 — RNA starting amounts vs library amplification, number cycles used, and concentration of washed libraries prior to pooling. [file Image2.TIF]

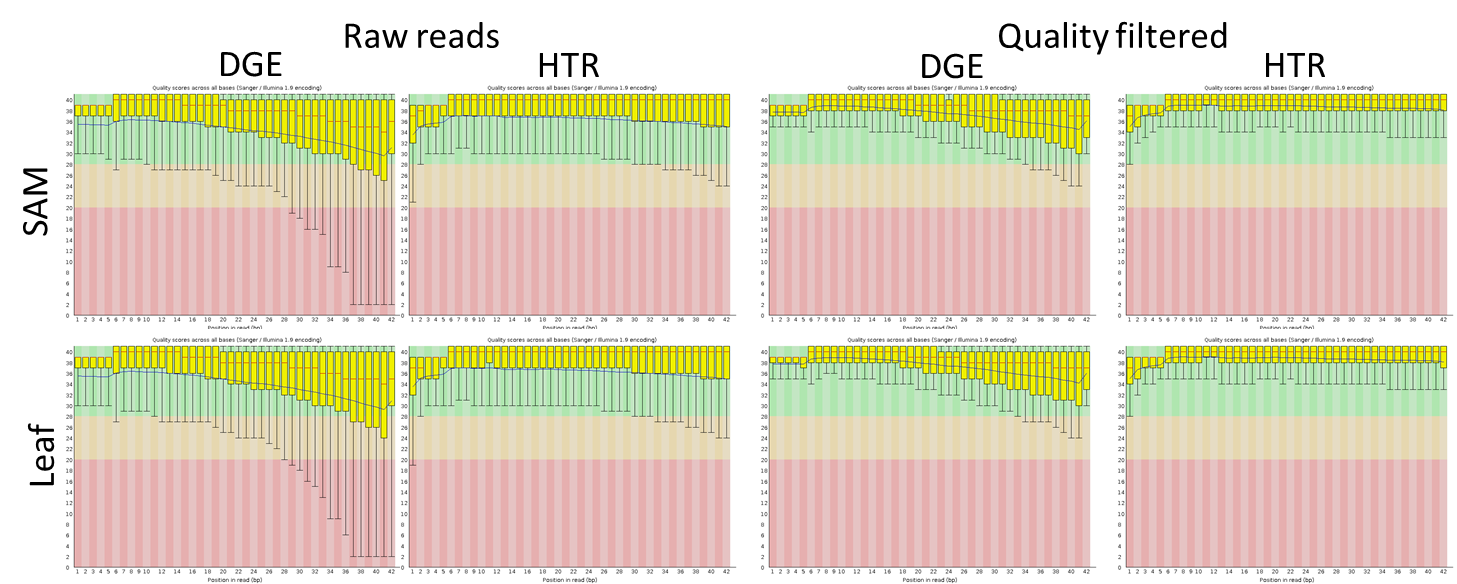

Supplement: Supplementary Figure 3 — Pre and post quality filtering PHRED scores for DGE and HTR libraries used in this study. [file Image3.TIF]

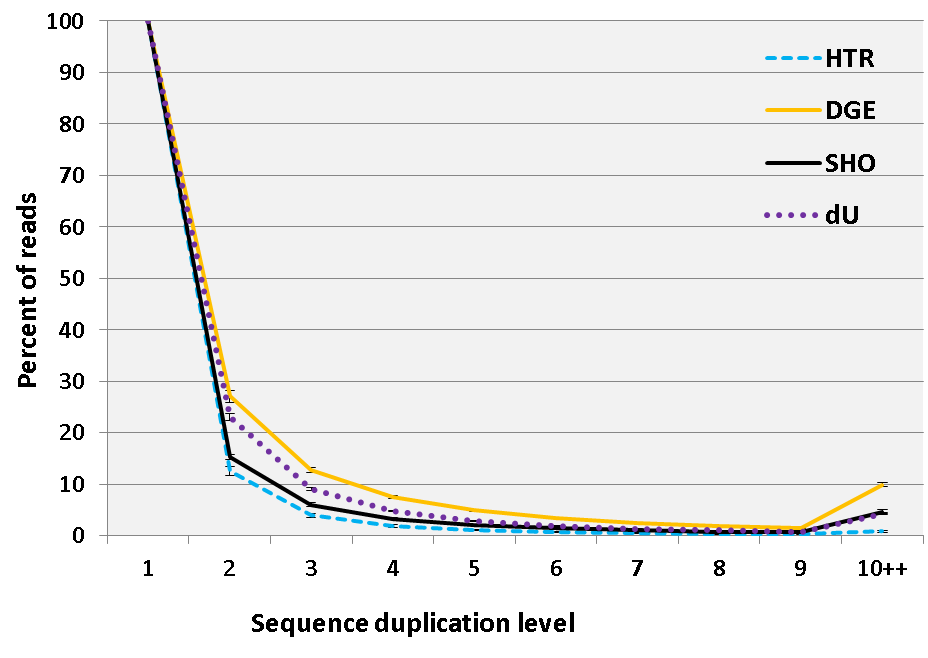

Supplement: Supplementary Figure 4 — Sequence duplication rates per million quality filtered reads. High throughput (HTR) 23.12 % (Black dashed), DGE 66.15% (Orange solid), Shotgun (SHO) 53.63% (Yellow solid), deoxy-Uracil marked (dU) 48.28% (Blue solid). [file Image4.TIF]

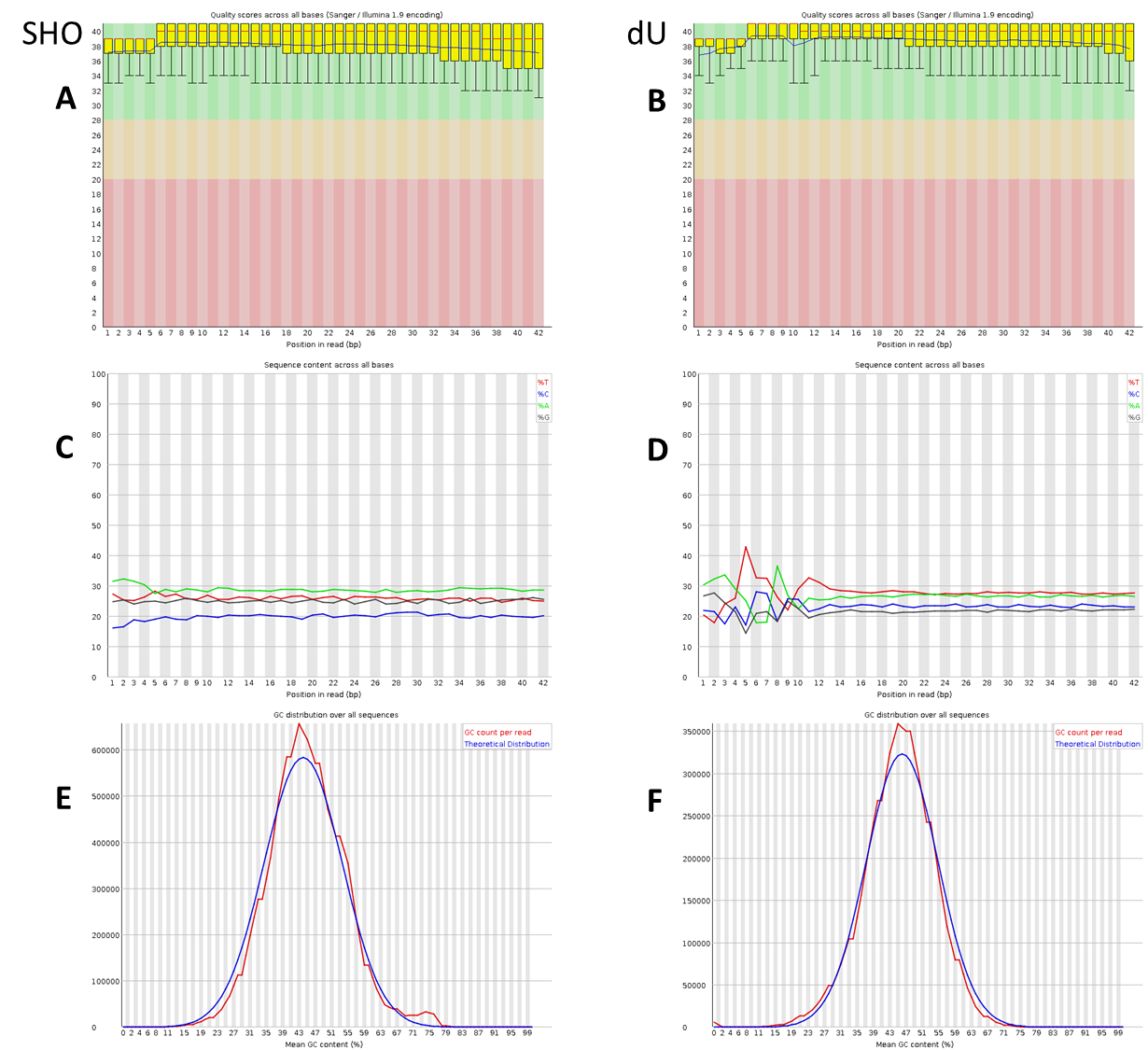

Supplement: Supplementary Figure 5 — FastQC analytics on filtered read information for additional strand specific library methods, Shotgun (SHO) (A,C,E), and deoxy-Uracil marked (dU) (B,D,F). Quality scores (A,B), Base composition (C,D), Percentage GC content (E,F). [file Image5.TIF]

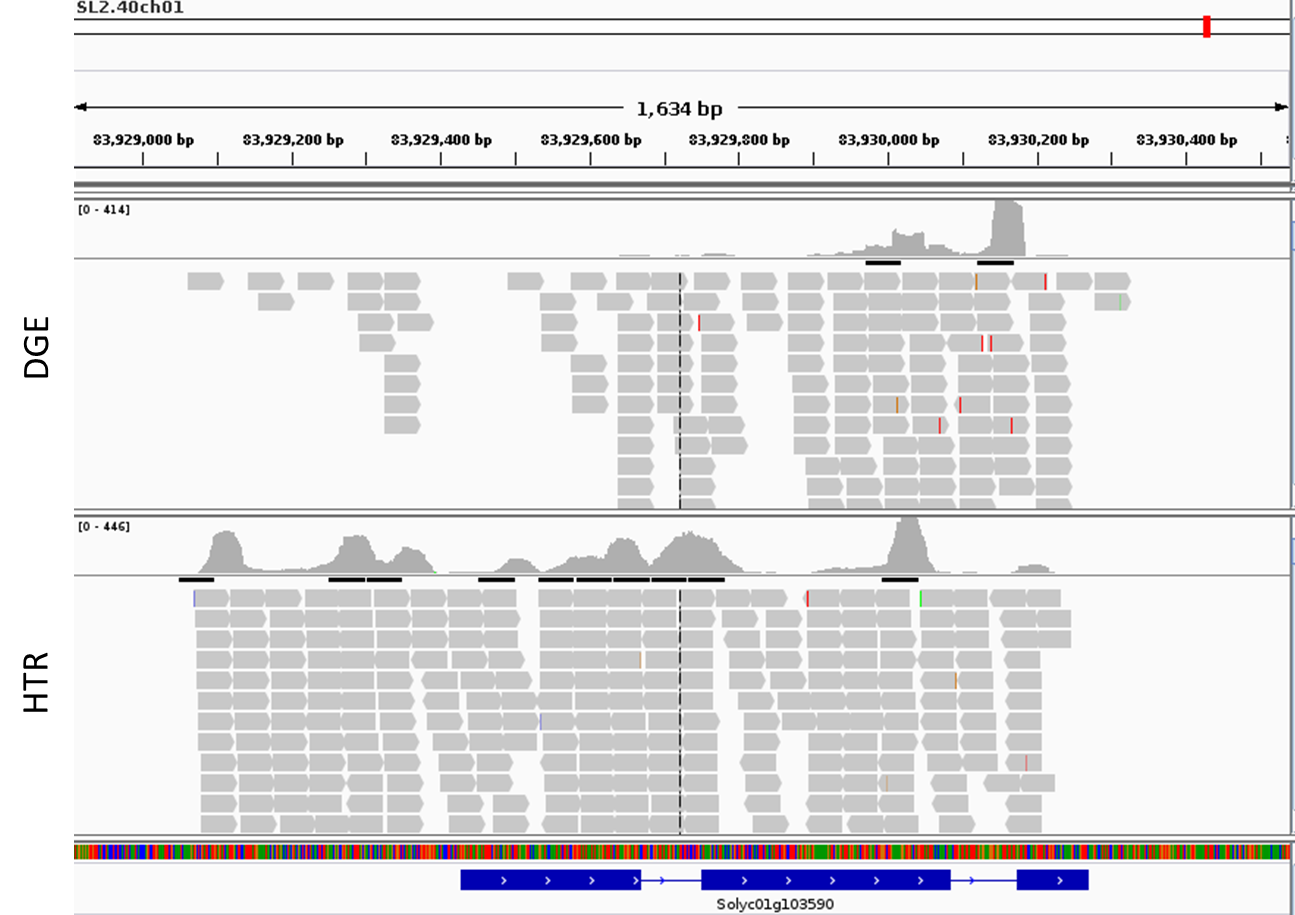

Supplement: Supplementary Figure 6 — Genomic mapping location of uniquely mapped reads in DGE and HTR. DGE reads show predominant localization to 3-prime of transcripts. [file Image6.TIF]

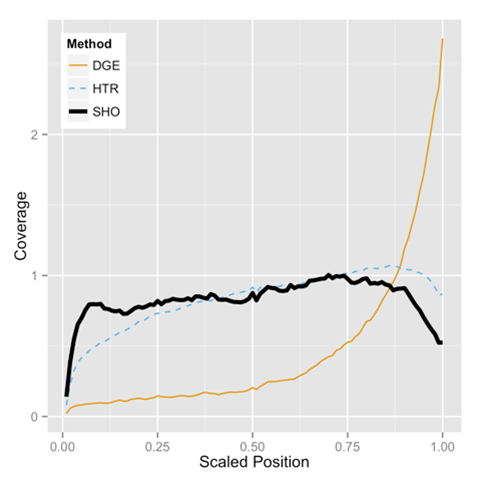

Supplement: Supplementary Figure 7 — Transcript coverage trace for SHO libraries. [file Image7.TIF]

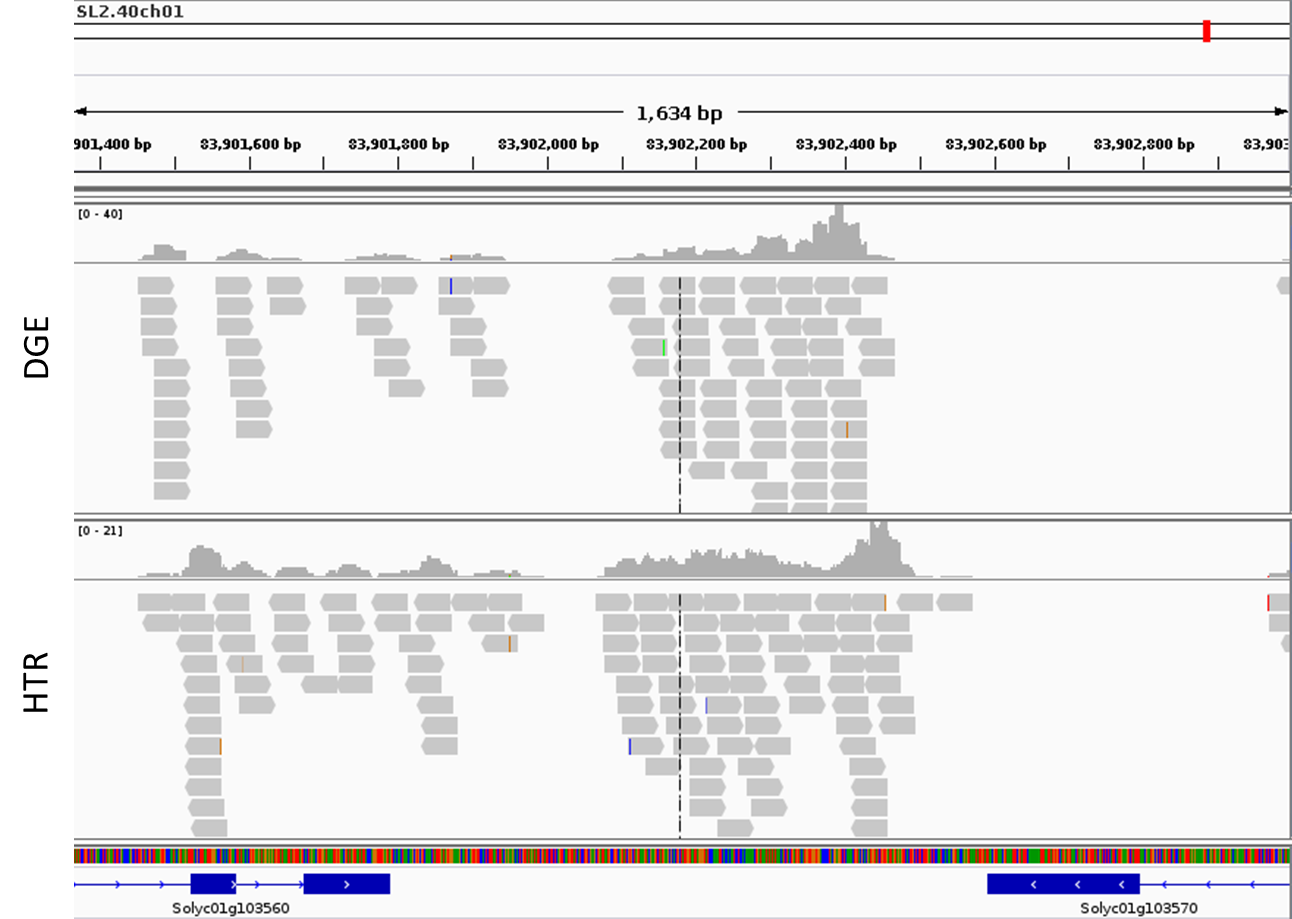

Supplement: Supplementary Figure 8 — Discrimination of read origin. DGE reads can be positively assigned to their transcript of origin when transcripts overlap or are in close proximity by strand specificity of the reads. [file Image8.TIF]

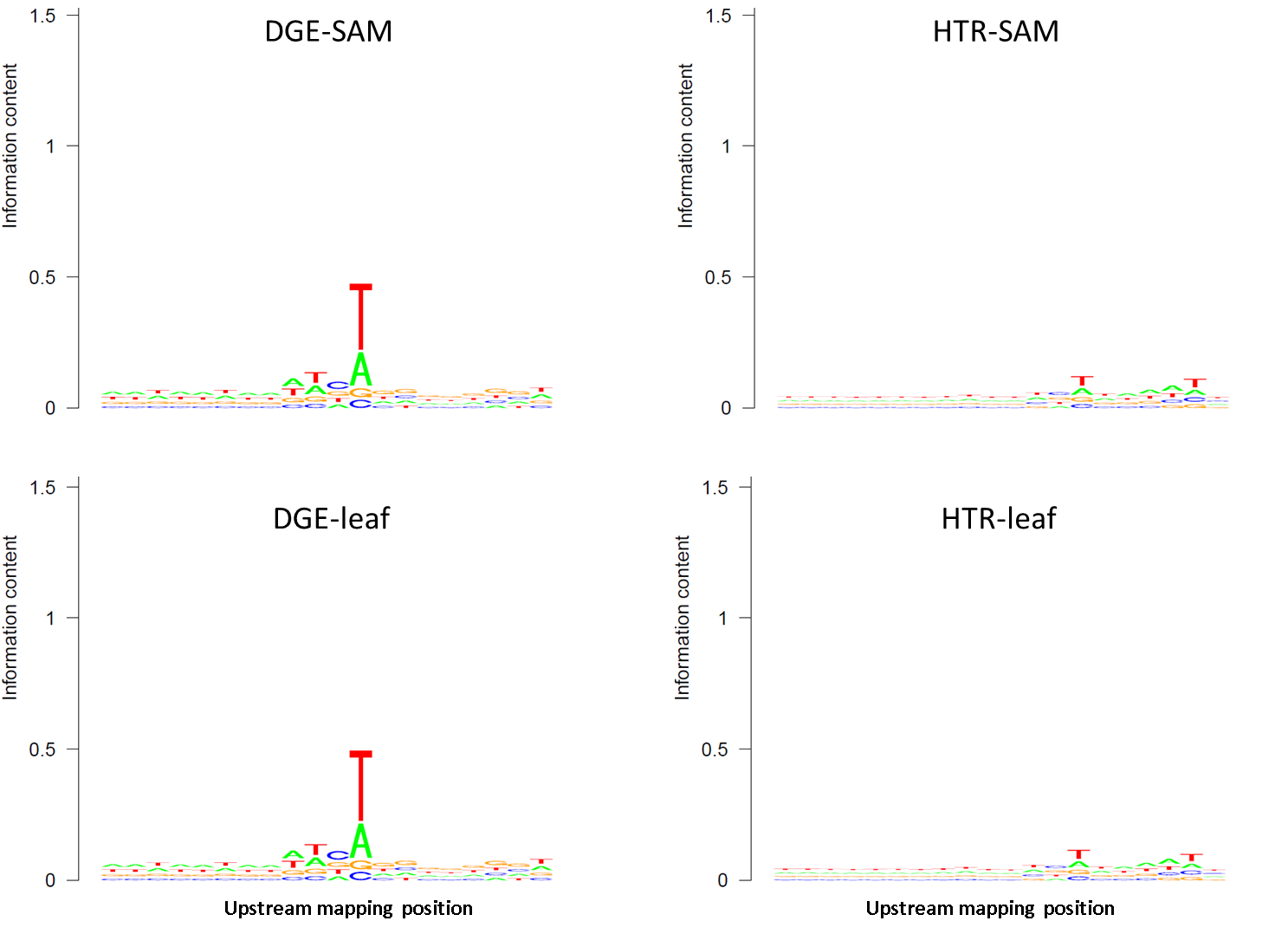

Supplement: Supplementary Figure 9 — Sequence logos displaying information content for 20 bases upstream of mapped reads. [file Image9.TIF]

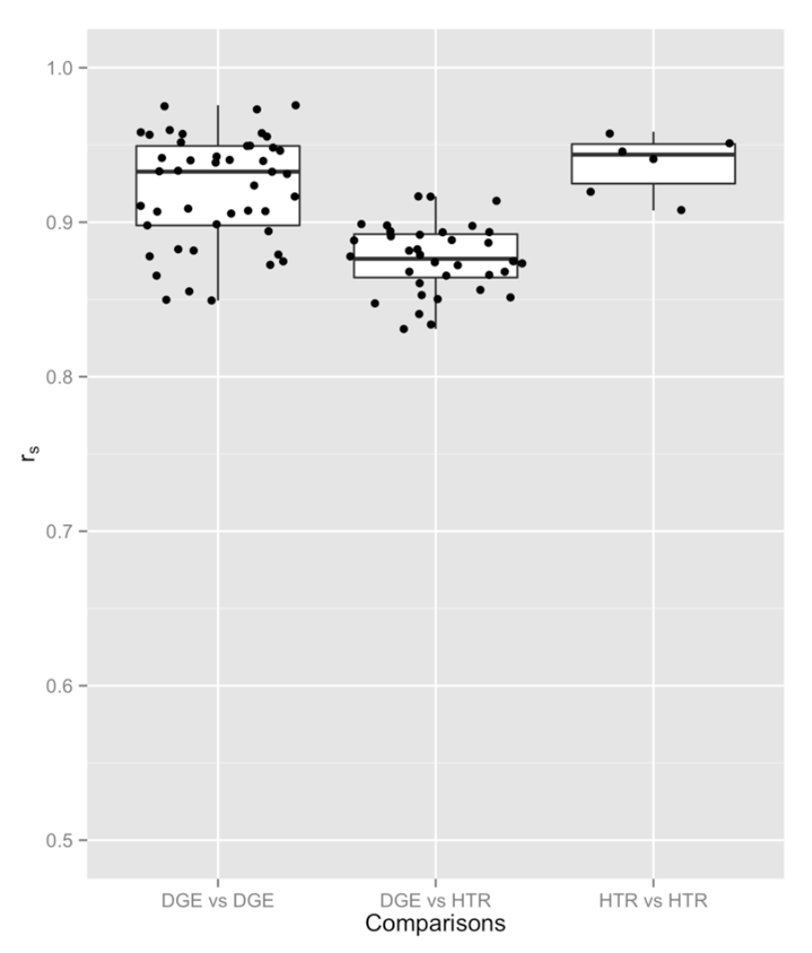

Supplement: Supplementary Figure 10 — Pairwise comparisons of differential gene expression shows higher correlation within each method than between methods. [file Image10.TIF]

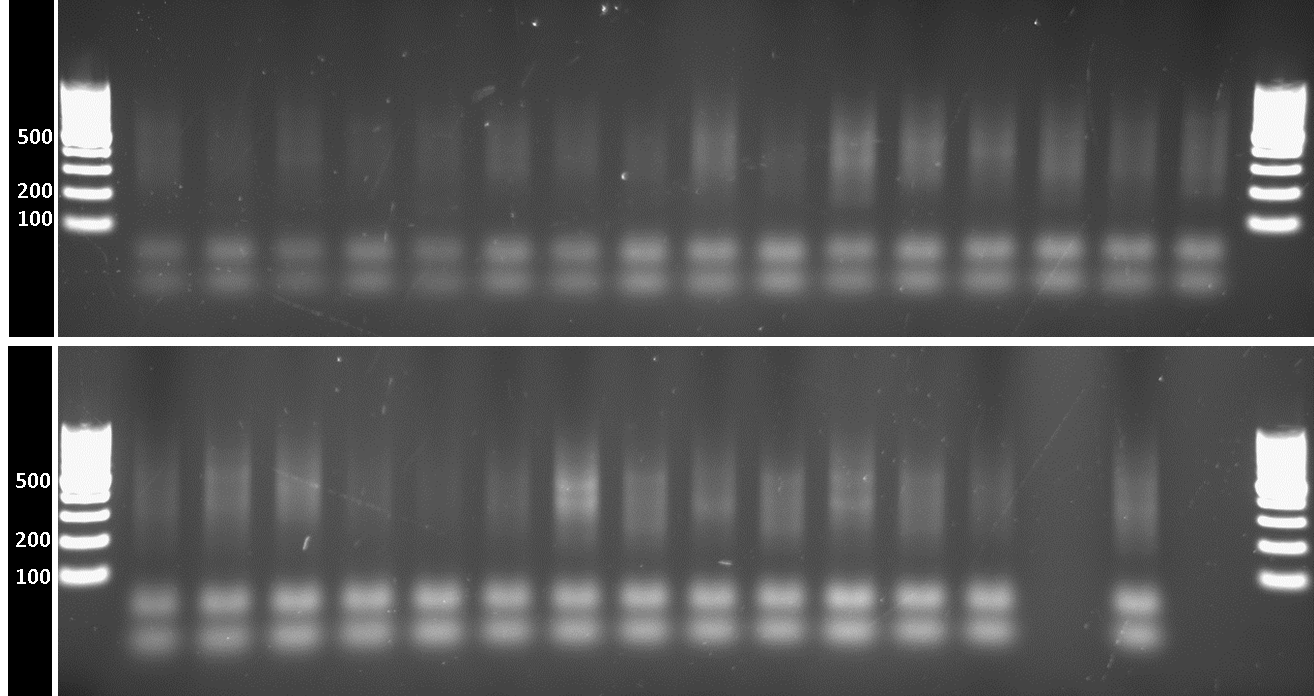

Supplement: Supplementary Figure 11 — Heterogeneous amplification from identical mRNA samples by single-stranded adapters containing barcode sequences near the 3-prime end. [file Image11.TIF]

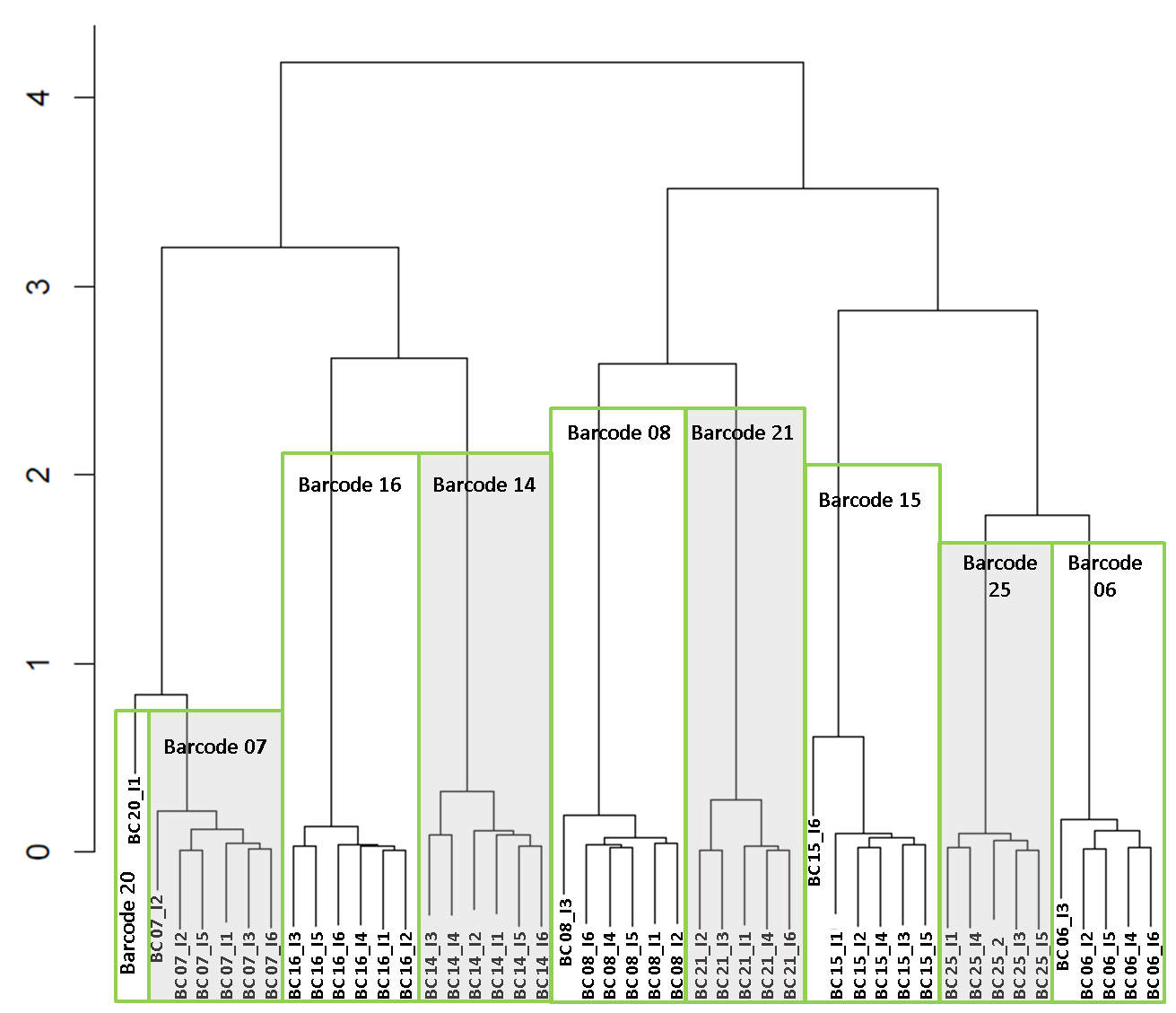

Supplement: Supplementary Figure 12 — Hierarchical clustering of library samples made with single stranded barcode containing adapters shows grouping only by barcode sequence. [file Image12.TIF]

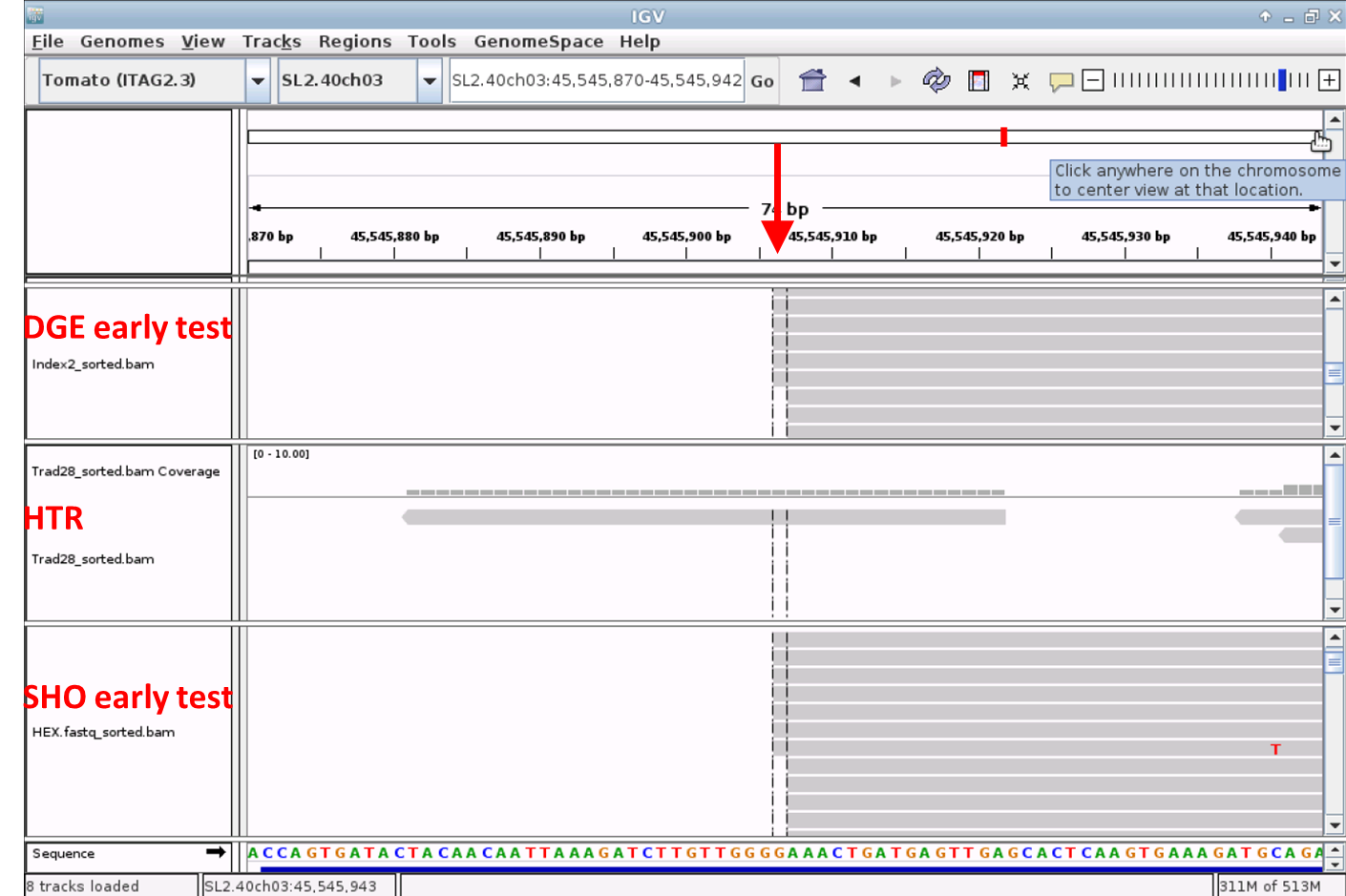

Supplement: Supplementary Figure 13 — Overrepresentation of reads mapping to positions containing Guanine repeats. [file Image13.TIF]

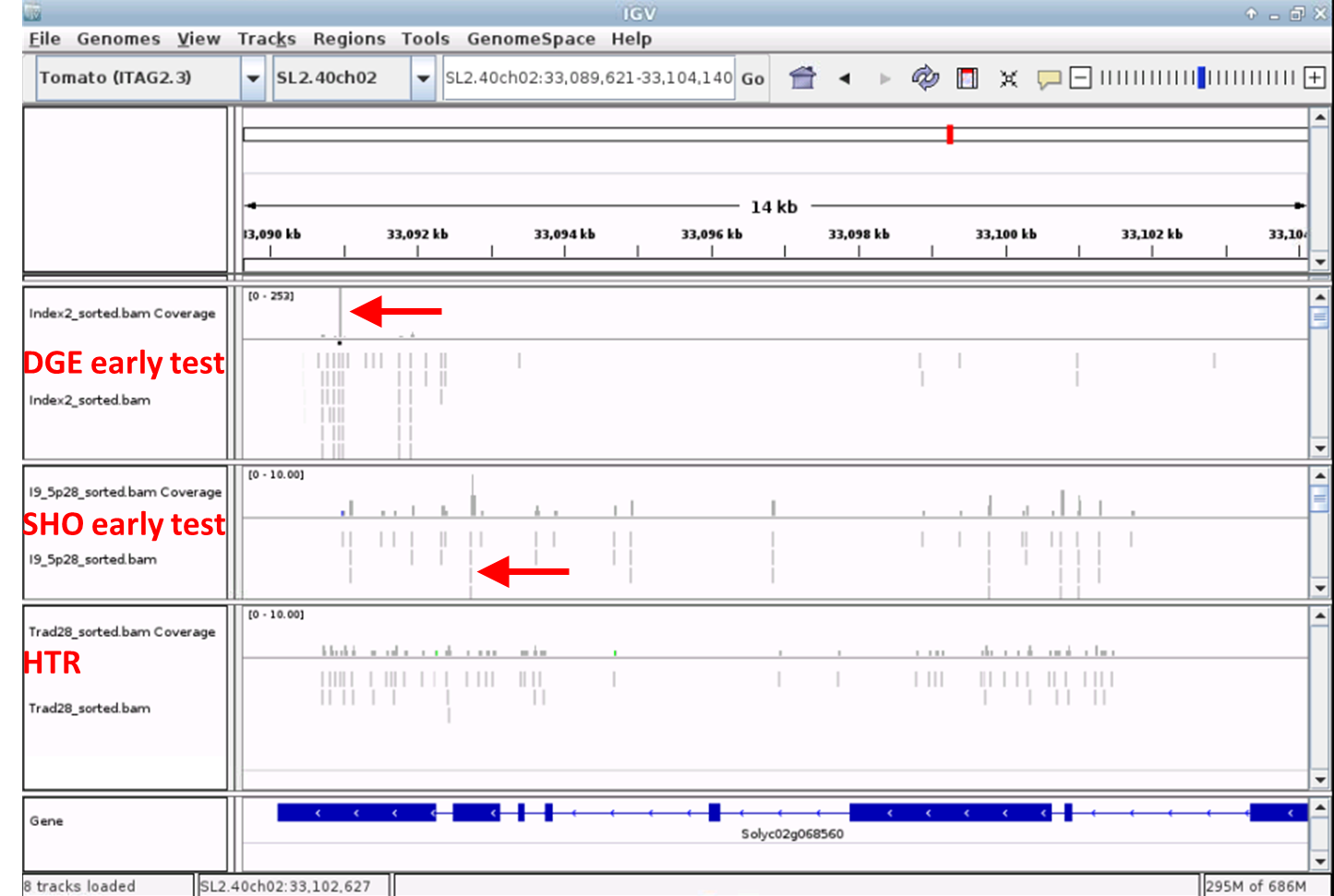

Supplement: Supplementary Figure 14 — Highly uneven distribution of mapping locations in libraries made with prototype adapters. [file Image14.TIF]

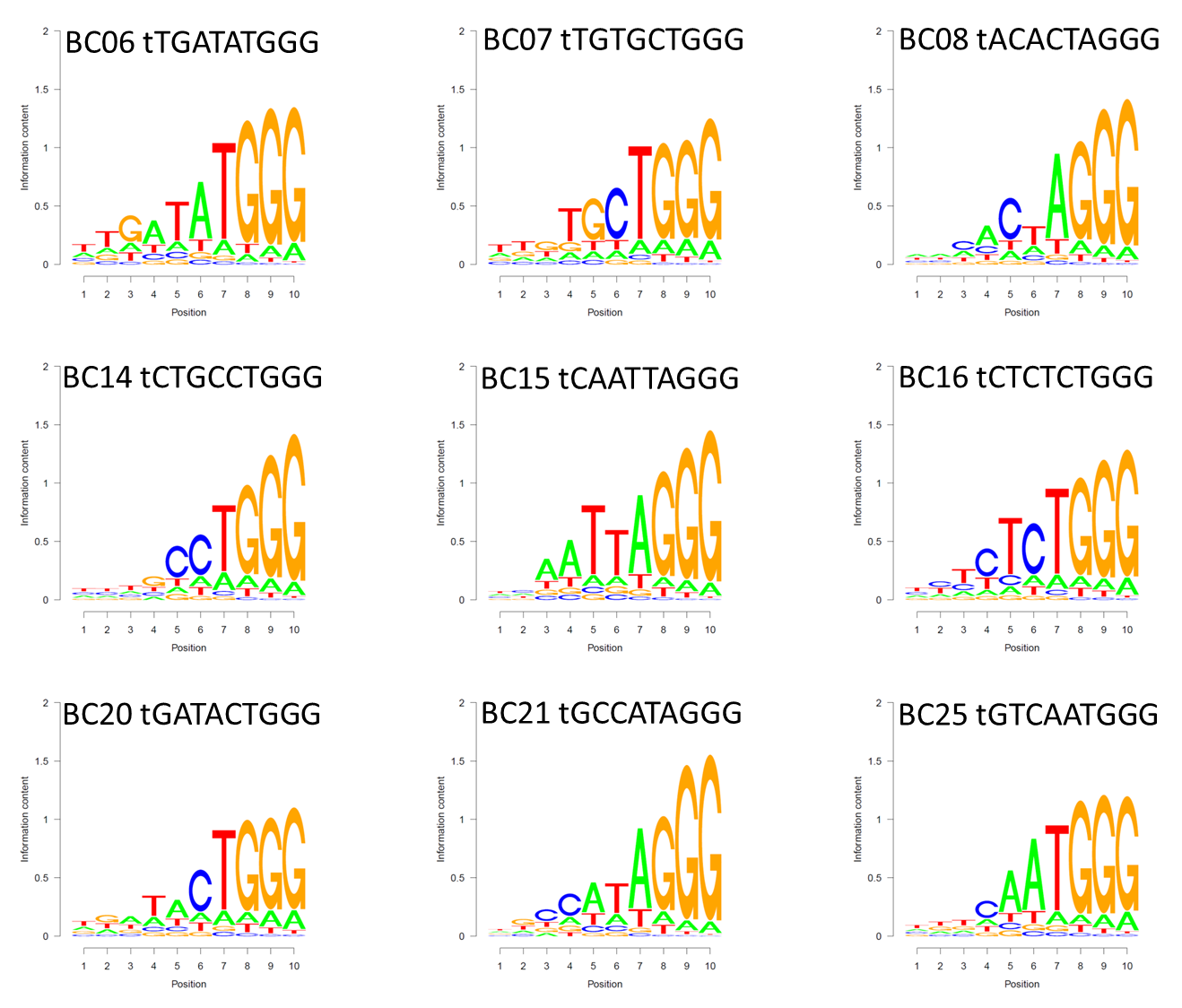

Supplement: Supplementary Figure 15 — Sequence information content for reads upstream of the first mapping nucleotide for the trimmed reads. [file Image15.TIF]
