# Supplementary material for: BrAD-seq: Breath Adapter Directional sequencing: a streamlined, ultra-simple and fast library preparation protocol for strand specific mRNA library construction
Source: Front Plant Sci. 2015 May 22;6:366. doi: 10.3389/fpls.2015.00366 (PMC4441129; doi:10.3389/fpls.2015.00366)
Supplement: Supplementary file 16 [file Table2.DOCX]

**Supplementary tables for: BrAD-seq: Breath Adapter Directional sequencing: a streamlined, ultra-simple and fast library preparation protocol for strand specific mRNA library construction.**

Brad Townsley**^1^**, Michael F. Covington**^1^**, Yasunori Ichihashi**^1,2,^**, Kristina Zumstein and Neelima Sinha**^1,*^**

University of California at Davis, Department of Plant Biology, Davis, California, United States

^2^ Present address: RIKEN Center for Sustainable Resource Science, Yokohama, Kanagawa, 230-0045 Japan

*** Correspondence:** Corresponding Author, University of California at Davis, Department of Plant Biology, One Shields Avenue, Davis, California, 95616, United States.

[nrsinha@ucdavis.edu](mailto:nrsinha@ucdavis.edu)

**Supplementary table 1** | Oligonucleotides

*See attached supplementary table 1 spreadsheet

**Supplementary table 2 |** User defined parameters for scripts used in this study.

| fastx_trimmer | -f 9 -Q 33 |
| --- | --- |
| trimFastqQuality.py | 20 35 |
| read_N_remover.py |  |
| adapterEffectRemover.py | 41 |
| Bowtie: |  |
| non-strand specific, non-uniquely mapped | -a --best --strata -v 1 -p 4 --sam --tryhard |
| non-strand specific, uniquely mapped | -a --best --strata -m 1 -v 1 -p 4 --sam --tryhard |
| strand specific, uniquely mapped | -a --best --strata --norc -m 1 -v 1 -p 4 --sam --tryhard |
|  |  |

**Supplementary table 3 |** R-squared values for all pairwise replicate sample comparisons log2 normalized read counts.

| Mean | **HTR leaf** | L_HTR_A4 | L_HTR_B5 | L_HTR_C6 | L_HTR_D7 |  |  |  |
| --- | --- | --- | --- | --- | --- | --- | --- | --- |
| 0.9277 | L_HTR_A4 |  |  |  |  |  |  |  |
|  | L_HTR_B5 | 0.9283 |  |  |  |  |  |  |
|  | L_HTR_C6 | 0.9307 | 0.9201 |  |  |  |  |  |
|  | L_HTR_D7 | 0.9320 | 0.9229 | 0.9324 |  |  |  |  |
|  |  |  |  |  |  |  |  |  |
| Mean | **HTR SAM** | S_HTR_A5 | S_HTR_A6 | S_HTR_B6 | S_HTR_B7 |  |  |  |
| 0.9064 | S_HTR_A5 |  |  |  |  |  |  |  |
|  | S_HTR_A6 | 0.9111 |  |  |  |  |  |  |
|  | S_HTR_B6 | 0.9086 | 0.9293 |  |  |  |  |  |
|  | S_HTR_B7 | 0.9209 | 0.8887 | 0.8797 |  |  |  |  |
|  |  |  |  |  |  |  |  |  |
| Mean | **DGE Leaf** | L_DGE_A4 | L_DGE_B5 | L_DGE_C6 | L_DGE_D7 | L_DGE_E7 | L_DGE_E8 | L_DGE_F1 |
| 0.9523 | L_DGE_A4 |  |  |  |  |  |  |  |
|  | L_DGE_B5 | 0.9599 |  |  |  |  |  |  |
|  | L_DGE_C6 | 0.9605 | 0.9600 |  |  |  |  |  |
|  | L_DGE_D7 | 0.9614 | 0.9603 | 0.9607 |  |  |  |  |
|  | L_DGE_E7 | 0.9572 | 0.9553 | 0.9519 | 0.9581 |  |  |  |
|  | L_DGE_E8 | 0.9541 | 0.9541 | 0.9516 | 0.9578 | 0.9657 |  |  |
|  | L_DGE_F1 | 0.9360 | 0.9328 | 0.9347 | 0.9405 | 0.9429 | 0.9433 |  |
|  |  |  |  |  |  |  |  |  |
| Mean | **DGE SAM** | S_DGE_A5 | S_DGE_A6 | S_DGE_B7 | S_DGE_C7 | S_DGE_D8 | S_DGE_E1 | S_DGE_F2 |
| 0.9582 | S_DGE_A5 |  |  |  |  |  |  |  |
|  | S_DGE_A6 | 0.9591 |  |  |  |  |  |  |
|  | S_DGE_B7 | 0.9564 | 0.9599 |  |  |  |  |  |
|  | S_DGE_C7 | 0.9567 | 0.9565 | 0.9518 |  |  |  |  |
|  | S_DGE_D8 | 0.9588 | 0.9594 | 0.9524 | 0.9608 |  |  |  |
|  | S_DGE_E1 | 0.9564 | 0.9590 | 0.9522 | 0.9615 | 0.9631 |  |  |
|  | S_DGE_F2 | 0.9575 | 0.9614 | 0.9557 | 0.9594 | 0.9617 | 0.9623 |  |
|  |  |  |  |  |  |  |  |  |
| Mean | **dU** | dU_1 | dU_2 | dU_3 |  |  |  |  |
| 0.9564 | dU_1 | dU_2 | dU_3 |  |  |  |  |  |
|  | dU_2 | 0.9564 |  |  |  |  |  |  |
|  | dU_3 | 0.9582 | 0.9545 |  |  |  |  |  |
|  |  |  |  |  |  |  |  |  |
| Mean | **SHO** | SHO_1 | SHO_2 | SHO_3 |  |  |  |  |
| 0.92252 | SHO_1 |  |  |  |  |  |  |  |
|  | SHO_2 | 0.926926 |  |  |  |  |  |  |
|  | SHO_3 | 0.920931 | 0.919703 |  |  |  |  |  |
