# Supplementary material for: BrAD-seq: Breath Adapter Directional sequencing: a streamlined, ultra-simple and fast library preparation protocol for strand specific mRNA library construction
Source: Front Plant Sci. 2015 May 22;6:366. doi: 10.3389/fpls.2015.00366 (PMC4441129; doi:10.3389/fpls.2015.00366)
Supplement: Supplementary file 17 [file DataSheet1.DOCX]

***Supplementary Methods 1: Non-strand specific mRNA libraries***

**BrAD-seq: Breath Adapter Directional sequencing: a streamlined, ultra-simple and fast library preparation protocol strand specific mRNA library construction.**

**mRNA fragmentation, 3-prime adapter priming and cDNA synthesis**

mRNA fragmentation was accomplished using magnesium ions at elevated temperature (Supplementary Figure 1). Priming for cDNA synthesis reaction was carried out in a single reaction mixture for Strand Specific-DGE, Strand Specific-RND, and non-Strand Specific libraries were fragmented in a reaction containing 1.5 μl 5X RT buffer (Thermo scientific, Cat. # EP0441), .5 μl of Invitrogen Random Primers (Invitrogen, Cat. # 48190-011) and 8.0 μl of the sample mRNA in a total reaction volume of 10 μl. Mixtures were spun down and incubated in a thermocycler. The following oligonucleotides and thermocycler programs were used for each library type.

**CNV**: Invitrogen random primers (Invitrogen, Cat. # 48190-011) (25°C 1 second, 94°C 1.5 min, 4°C 5 min, 20°C hold).

**cDNA synthesis**

cDNA was synthesized by addition of 5 μl of the following reaction mixture to the fragmented and primed mRNA: 1.5 μl 5X Thermo Scientific RT buffer (Thermo scientific, Cat. # EP0441), 1.5 μl 0.1M Dithiothreitol (DTT), 1 μl H2O, 0.5 μl 25mM dNTPs (Thermo Scientific, Cat. # R1121), 0.5 μl RevertAid RT enzyme (Thermo scientific, Cat. # EP0441) (total reaction volume 15 μl). The reaction mixture was set up at room temperature and placed in a thermocycler running the following program: (25°C 10 min, 42°C 50 min, 50°C 10 min, 70°C 10 min, 4°C hold). cDNA was cleaned and size-selected prior to “breath capture” or second strand synthesis by addition of 5 μl 50 mM EDTA pH 8.0 and 30 μl Agencourt AMPure XP beads (Beckman, Cat. # A63881) to each sample and mixed by pipetting. After 5 minutes, samples were placed on a magnetic tray, supernatant was removed, and pellets were washed twice with 300 μl 80% ethanol without pellet disruption. Residual ethanol was removed with 20-μl pipette tip and samples were allowed to air-dry until no visible traces of liquid were detectable.

**Second strand synthesis, end repair and A-tailing (non-strand specific)**

Second strand synthesis was accomplished by adding 5 μl of the following reaction mixture directly the cDNA reaction: 1.5 μl H2O, 0.4 μl 25mM dNTPs, 1 μl PolI (Thermo Scientific, Cat. # EP0041), 0.1 μl RNaseH (New England BioLabs, Cat. # M0297S ), 0.4 μl End repair mix (New England BioLabs, Cat # E6050L), 0.2 μl Taq polymerase (New England BioLabs, Cat. # M0273L), 1.4 μl End Repair Buffer (New England BioLabs, Cat. # E6050L). The reaction was set up at room temperature and incubated in a thermalcycler at running the following program: (16C 20m, 20C 20m, 72C 20m, 4C Hold). Cleanup was done as in previous steps using 30 μl Ampure XP beads and 2 300 μl 80% ethanol was steps.

**Adapter ligation (non-strand specific)**

Adapter ligation was done by adding to the cDNA containing pellet 3 μl of pre annealed 1 μM universal ligation adapter consisting of 1 μM each PE1-lig (5’-CACTCTTTCCCTACACGACGCTCTTCCGATCT-3’) and ILL-lig (5’-P-GATCGGAAGAGCACACGTCTGAACTCCAGTCAC-3’) which contains a 5-prime phosphate group. The universal ligation adapter was annealed as described above. To the hydrated pellet 7 μl of the following reaction mixture was added: 1.75 μl H2O, 5.0 μl 2X Rapid T4 ligase buffer (Enzymatics, Cat. # L603-HC-L), 0.25 μl E. coli DNA ligase (New England Biolabs, Cat. # M0348L). The reaction was mixed by pipetting and allowed to stand at room temperature for 15 minutes. Cleanup and size selection was done by adding 10 μl of 50 mM EDTA and 25 μl of ABR to each sample and treated as in the previous step. Pellets were re-suspended in 22 μl 10mM Tris pH 8.0, allowed to stand 1 minuet and place on magnetic tray. Supernatant was transferred without beads to fresh strip tubes and stored at -20 prior to enrichment.
