# Supplementary material for: BrAD-seq: Breath Adapter Directional sequencing: a streamlined, ultra-simple and fast library preparation protocol for strand specific mRNA library construction
Source: Front Plant Sci. 2015 May 22;6:366. doi: 10.3389/fpls.2015.00366 (PMC4441129; doi:10.3389/fpls.2015.00366)
Supplement: Supplementary file 18 [file DataSheet2.DOCX]

***Supplementary Methods 2: Detailed protocol***

**BrAD-seq: Breath Adapter Directional sequencing: a streamlined, ultra-simple and fast library preparation protocol for strand specific mRNA library construction.**

**Contents**

**1: Nucleic acid isolation protocols**

**1.1: Streptavidin bead mRNA isolation**

**1.2 DNA isolation (LBB DNA preps)**

**2: RNA fragmentation and priming (strand specific and non-specific)**

**3: cDNA Synthesis (strand specific and non-specific)**

**4: Adapter annealing**

**5: 5-prime adapter sequence addition (strand-specific only)**

**6: Second strand synthesis and end preparation (Conventional non-strand specific only)**

**6.1: Non-strand specific mRNA libraries**

**6.2: Double-stranded DNA input**

**6.3: Single stranded DNA input**

**7: Enrichment and adapter extension**

**8: Final library cleanup**

**1.1: Streptavidin bead mRNA isolation**


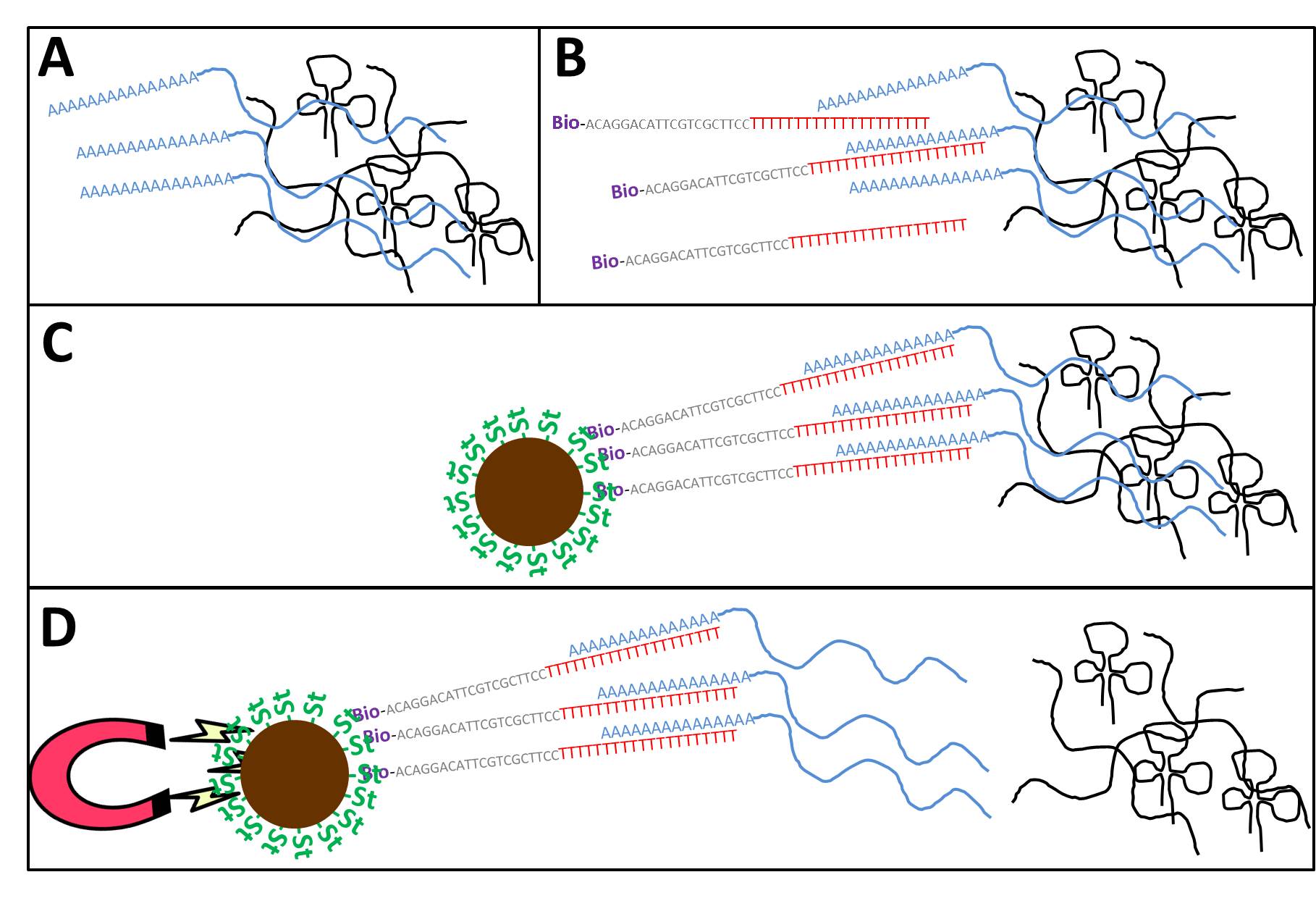


mRNA constitutes only a few percent of the total amount of RNA in a living cell, the rest of the RNA is composed of ribosomal RNAs, transfer RNAs, splicosomal RNAs and numerous other non-mRNA species. In eukaryotic cells, mRNAs differ from other forms of RNA in the cell because at their 3-prime end a “poly A tail”, a long stretch of Adenine ribonucleotides are added during transcription (A). When an oligonucleotide containing a long stretch of Thymine nucleotides is added to the tissue lysate they are able to base-pair and form a strong double stranded region (B). A biotin molecule is attached to the 5-prime end of the oligo during synthesis because of the strong bond that is formed between biotin and the Streptavidin protein when they come into contact, one of the strongest non-covalent bonds in nature. When magnetic Streptavidin coated beads are added to the tissue lysate, the biotin molecule attached to the poly T oligo base-paired with an mRNA molecule are bound by the Streptavidin linking the mRNA to the magnetic bead (C). When a magnetic field is applied to the beads, the mRNA remains associated with the beads as they pellet allowing any other RNAs, DNA and other contaminants to be washed away (D).

Things to remember:

● When you are working with RNA exorcise extra diligence and cleanliness. Chances are whatever assay or experiment you are going to be performing is highly sensitive to contamination so use a dedicated “clean” set of pipettors (i.e. not ones used for running gels and doing plasmid preps). Maintaining a clean work area, ensuring your gloves remain clean and avoiding sample splashing and touching the inside of tube lids during opening and closing is most of what is needed to ensure success. Filter tips are expensive totally unnecessary if good lab practices are observed.

● The stock solutions are common and used by everyone. Don’t risk contaminating them by sticking your pipettor into the bottles. If you have questions don’t be afraid to ask a grownup for help.

● The beads are what hold the mRNA you are isolating so make sure the beads have fully magnetized out of solution before removing the lysate and during the washing steps. Depending on the tissue you are using the lysate can be intensely colored and make it more difficult to tell so err on the side of caution. During the washes you can place white paper behind the tubes to be sure all of the beads have pelleted.


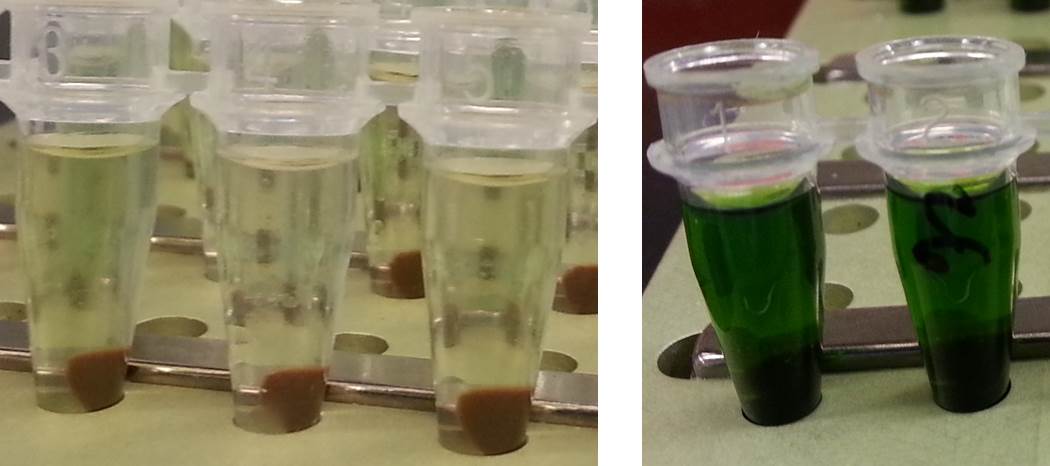


Stock solutions:

● 1 M Tris-HCl pH 8 (Store at room temperature)

● 8 M LiCl (Store at room temperature)

● 500 mM EDTA pH 8 (Store at room temperature)

● 5% W/V SDS (Store at room temperature)

● 500 mM DTT (make 500 μl aliquots and store at -20 C)

● Antifoam A (Store at room temperature)

● 5 M NaCl (Store at room temperature)

● 14.3 M 2-Mercaptoethanol (Store at 4 C)

Reagents:

● NEB Streptavidin magnetic beads (Store at 4 C and keep on ice prior to use. Swirl repeatedly before use to re-suspend beads fully before each use.)

Catalog #: S1420S Size: 5 ml Concentration: 4 mg/ml

● 12.5 μm Biotin-linker-polyT oligo. (Store at -20 C. Return to -20 C promptly after each use.)

Oligo sequence:

5’-Biotin-ACAGGACATTCGTCGCTTCCTTTTTTTTTTTTTTTTTTTT-3’

Prepare as needed by adding 12.5 μl from the 100 μm stock to 87.5 μl RNAse-free H20. Vortex thoroughly.

Working solutions:

● Lysis/binding buffer (**LBB**) (Store at 4 C and warm up to RT before use by placing at 37 C for several minutes)

Solution components stock solutions volumes for 50 ml

100 mM Tris-HCl 1 M pH 8 5 ml

1M LiCl 8 M 6.25 ml

10 mM EDTA 500 mM pH 8 1 ml

1% SDS (or LiDS) 5% w/v 10 ml

5 mM DTT .5 M 500 μl

Antifoam A 750 ul

RNAse-free H2O to 50 ml

Immediately before adding to ground tissue add 5 μl/ml 2-Mercaptoethanol.

Ensure salt crystals are fully dissolved and Antifoam A is fully homogenized in solution prior to each use.

● Washing Buffer A (**WBA**) (Store at 4 C and keep on ice prior to use)

Solution components stock solutions volumes for 50 ml

10 mM Tris-HCl 1 M pH 8 500 μl

150 mM LiCl 8 M 940 μl

1 mM EDTA 500 mM pH 8 100 μl

0.1% SDS 5% w/v 500 μl

RNAse-free H2O to 50 ml

● Washing Buffer B (**WBB**) (Store at 4 C and keep on ice prior to use)

Solution components stock solutions volumes for 50 ml

10 mM Tris-HCl 1 M pH 8 500 μl

150 mM LiCl 8 M 940 μl

1 mM EDTA 500 mM pH 8 100 μl

RNAse-free H2O to 50 ml

● Low-salt Buffer (**LSB**) (Store at 4 C and keep on ice prior to use)

Solution components stock solutions volumes for 50 ml

10 mM Tris-HCl 1 M pH 8 500 μl

150 mM NaCl 5 M 1.5 ml

1 mM EDTA 500 mM pH 8 100 μl

RNAse-free H2O to 50 ml

● 10 mM Tris-HCl pH 8 (Store at room temperature)

Solution components stock solutions volumes for 50 ml

10 mM Tris-HCl 1 M pH 8 1 ml

RNAse-free H2O to 50 ml

● 1 M (1000 mM) 2-Mercaptoethanol (**freeze immediately at -20 between uses and re-make frequently**)

Solution components stock solutions volumes for 100 μl

1 M 2-Mercaptoethanol 14.3 M 7 μl

RNAse-free H2O 93 μl

● RNA elution buffer 10 mM Tris-HCL, 1mM 2-Mercaptoethanol (**make fresh each time**)

Solution components stock solutions volumes for 1 ml

10 mM Tris-HCl 10 mM 999 μl

1 M 2-Mercaptoethanol 1 M 1 μl

**Procedure:**

This procedure uses 200 μl of tissue lysate containing up to 20 mg of tissue.

Tissue preparation (For samples weighing more than 10 mg)

For samples weighing 1-5 mg use the small sample modifications to the steps below as noted.

● Grind in liquid N2 or bead beat sample tissue to fine powder prior to adding LBB.

● Allow samples to “warm” up on dry ice or in -80 to allow all liquid N2 to evaporate from samples before proceeding. (**Prevents exploding tubes**)

● For each 100 mg of finely ground tissue add 1 ml of LBB and vortex to homogenize

Small samples: Add 105 μl of LBB and vortex to homogenize

*Keep the samples in a deep Styrofoam box with lid containing dry ice and remove the lid only to remove each sample.*

● If any fragments of tissue are visible, place tubes in room temperature bead beater block and bead beat for an additional 1 minute to fully homogenize tissue lysate. (This is particularly important for small tissue samples)

● Allow to sit at room temperature for 5 minutes

● Invert each tube several times and place in centrifuge for 10 minutes at maximum speed.

● Carefully transfer supernatant to fresh tubes avoiding transfer of tissue debris.

mRNA capture:

● For each sample place 1 μl of 12.5 μM biotin-20nt-20T oligo into each well of strip tube or plate.

Small samples: use .5 μl of 12.5 μM biotin-20nt-20T oligo.

● Add 200 μl of lysate to each well and mix well by pipetting. Store the remainder of tissue lysate at -80 C for future use.

Small samples: Use all of lysate volume. Incubation time can be increased if it makes you feel better.

● Allow to stand at Room temperature for 10 mins.

*--prepare Streptavidin beads while you wait—*

● gently re-suspend streptavidin beads in their container before proceeding.

● For each sample place 20 μl of NEB magnetic Streptavidin beads in separate tubes.

Small samples: use 10 μl of Streptavidin beads.

● Place on magnetic tray and remove supernatant.

● Re-suspend beads in 100 μl of LBB.

● Place on magnetic tray and remove supernatant.

● Re-suspend bead pellets with transferred lysate containing Biotin-20nt-PolyT oligo

● Place on agitator 10 min at room temperature.

● Briefly spin strips to remove lysate from strip lids.

● Place on magnetic tray and remove supernatant.

*If DNA is to be isolated from these samples then transfer mRNA depleted lysate to .5 or 1.5 ml Epindorf tubes and set aside for LBB DNA preps.*

● Wash sequentially with 300 μl of cold WBA, WBB and LSB fully re-suspending pellet with each wash.

● Place on magnetic tray and remove supernatant.

● Re-suspend pellet in 16 μl RNA elution buffer (**Elution volume should be adjusted based on your needs**)

Small samples: Re-suspend pellet in 8 μl RNA elution buffer

● Heat to 80 C In thermocycler for 2 min and place immediately on magnetic rack.

● Transfer supernatant containing mRNA to fresh tubes, avoiding bead carryover.

*Tilting strip tubes helps minimize bead carryover. You may need to transfer mRNA samples to a second fresh tube if visible beads present.*

● Quantify using the nanodrop (optional) or Bioanalyzer (recommended for first trials of a new tissue) . Tomato leaf lysate should yield ~20 ng/μl

If the tissue and amount have been done before with good results there is no need to quantify

If you wish to do a secondary enrichment of your mRNA, there are sufficient unoccupied streptavidin molecules on the used beads to do so without using fresh beads. Simply wash the used beads with 200 μl Tris or RNAse-free H2O, add your mRNA sample to 200 μl of LBB and repeat the process.

*When working with small amounts of mRNA and particularly for non-DGE libraries which do not prime from poly-A tails, you may wish to first check some test mRNA samples on a bioanalyzer. When mRNAs are limiting non-specific interactions may occur. Selection of the most appropriate mRNA prep method for a new sample type early in an experiment can save time on troubleshooting and optimization later.


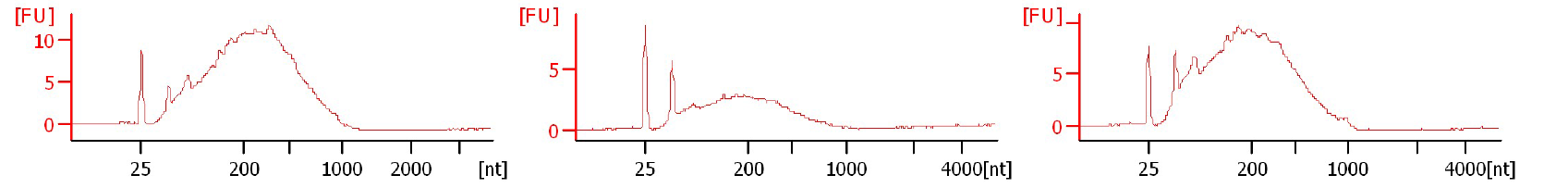


Bioanalyzer traces of mRNA isolations showing non-coding RNA carryover (above 25bp marker).

**1.2: DNA isolation (LBB DNA preps)**

DNA can be isolated from mRNA depleted lysate or LBB tissue lysate specifically intended for DNA isolation. LBB still requires addition of 2-Mercaptoethanol for DNA only extractions.

The precipitation of DNA with alcohol co-precipitates numerous other substances that can interfere with the re-suspension of DNA or subsequent uses. There are a couple of ways to minimize or remove these interfering substances. Since much of the co-precipitate is protein, a proteinase K treatment prior to precipitation can significantly reduce the amount of total precipitated material and increase the recovery of DNA from the pellet.

Stock solutions:

● 100% Isopropanol

● 100% Acetone

● 100% Ethanol

● 80% Ethanol

● 3M Ammonium acetate (or Sodium Acetate)

● Proteinase K storage buffer, 10 ml (Store at -20 C) **Optional**

Buffer components:

CaCl2 .03 g

1M Tris pH 8 100 μl

H20 4.9 ml

Glycerol 5.0 ml

● Proteinase K working stock, 10 mg/ml (Store at -20 C) **Optional**

Dissolve 10 mg of proteinase K in 1 ml of storage buffer.

Reagents:

● Promega Proteinase K

Catalog #: V3021 Size: 100 mg

**Procedure:**

● Add 5 μl of 10 mg/ml Proteinase K to each 200 μl of LBB tissue lysate. Mix well and incubate at 37 C for 30 minutes. (**Optional. Not usually needed**)

● To LBB lysate add 1 volume 100% Isopropanol

● Invert several times to mix completely, let stand 5 minutes and place in centrifuge on maximum for 5 minutes.

● Remove and discard supernatant without disturbing pellet.

● Add 400 μl of 100% Acetone to each tube. Let stand at room temperature for 5-10 minutes with occasional gentle inversion.

*You should be able to see acetone soluble substances dissolving into the solution and the acetone taking on the color of the pellet. You should be able to see when this process has gone as far as it is going to.*

● carefully remove supernatant avoiding disturbing pellet. The pellet may have become dislodged so be careful not to suck it up. Centrifuge if needed.

● Add 400 μl of 100% Acetone for second wash. Let stand at room temperature for 1-10 minutes with occasional gentle inversion. You will see when the pellet is fully de-colored.

● carefully remove supernatant avoiding disturbing pellet. Centrifuge 1 minute on maximum if it seems needed.

● Allow pellet to dry fully and add 100 μl 10 mM Tris-HCl pH 8. (Drying of Acetone occurs quickly.)

● Allow DNA to re-suspend into solution. Place at 65 C (Agarose oven or heat block) for 10 mins to speed the process and heat inactivate any carryover proteinase K. Gently flick the bottom of the tubes occasionally to homogenize the solution without grinding the pelleted junk into unmanageably small pieces.

*If Proteinase K is not used then allowing to sit at 4 C overnight re-suspends DNA very effectively without disturbing the pelleted junk.*

● Centrifuge samples on maximum for 1 minute.

● Transfer up to 100 μl of each sample to fresh .5 ml or 1.5 ml Epindorf tubes being careful to avoid chunks of pellet debris.

● **OPTIONAL** RNAse treatment. (**Not that important**)

There will be a significant amount of ribosomal RNA contained in the final DNA extract. This is generally not a problem but if it poses a specific problem for the intended purpose of the DNA then use RNAse I or **CERTIFIED** DNAse free RNAse A to degrade any RNA in the sample prior to the following precipitation step.

● Add 10 μl of 3M Ammonium acetate to each sample and mix.

*3M Sodium acetate should also be fine. An advantage of using Ammonium over Sodium is that excess Ammonia will evaporate during sample drying where Sodium will not.*

● Add 250 μl of 100% EtOH to each sample and mix by inverting several times and let stand 5 minutes.

● Centrifuge on maximum for 2-5 minutes.

*If you can see precipitate forming quickly then shorter centrifuge times are fine but if the amount of DNA is very small longer times are recommended.*

● Carefully remove and discard supernatant with a 200 μl pipette. The DNA pellet should be very clean and thus totally invisible so be careful to avoid the areas of the tube where the pellet would form. When the supernatant is removed the DNA should be as transparent as glass.

● Wash DNA pellet by adding 400 μl 80% EtOH to each tube and mix by inverting. Allow to stand 2 minutes with occasional inverting to remove excess Ammonium acetate.

● Centrifuge on maximum for 30 seconds.

● Carefully remove supernatant and dry samples in hood or speed-vac.

● Add 50-100 μl 10 mM Tris or H20 to each pellet to re-suspend DNA.

● Run 1 μl on an agarose gel to qualitatively verify the DNA quality.


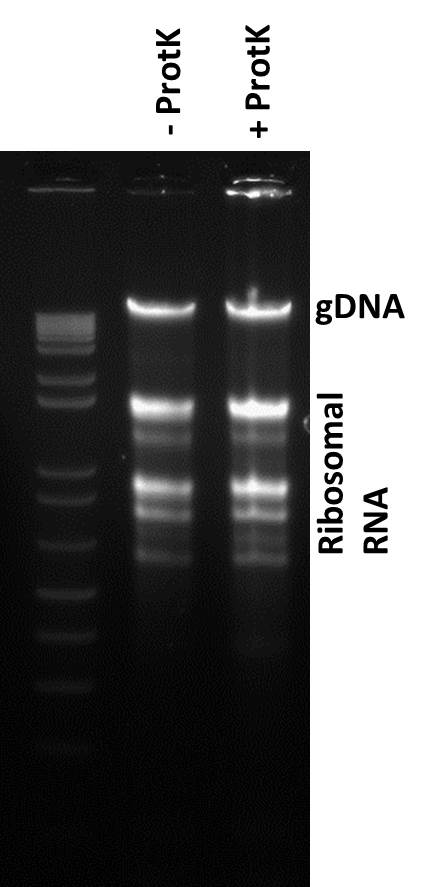


2 μl of 50 from a prep using 200 μl of tomato LBB lysate. Not treated with RNAse.

**2: RNA fragmentation and 3-prime adapter cDNA priming**

Magnesium ions in the first strand buffer are used to fragment the mRNA at high temperature, followed by priming of the 1^st^ strand cDNA by the appropriate adapters. All subsequent RNA library modules share this step, weather strand-specific 3-prime Digital Gene Expression (DGE), strand-specific non-DGE Shotgun (SHO) or Conventional (CNV) non-strand-specific RNA-seq libraries, each varies only in the priming oligo and the priming portion of the fragmentation program.


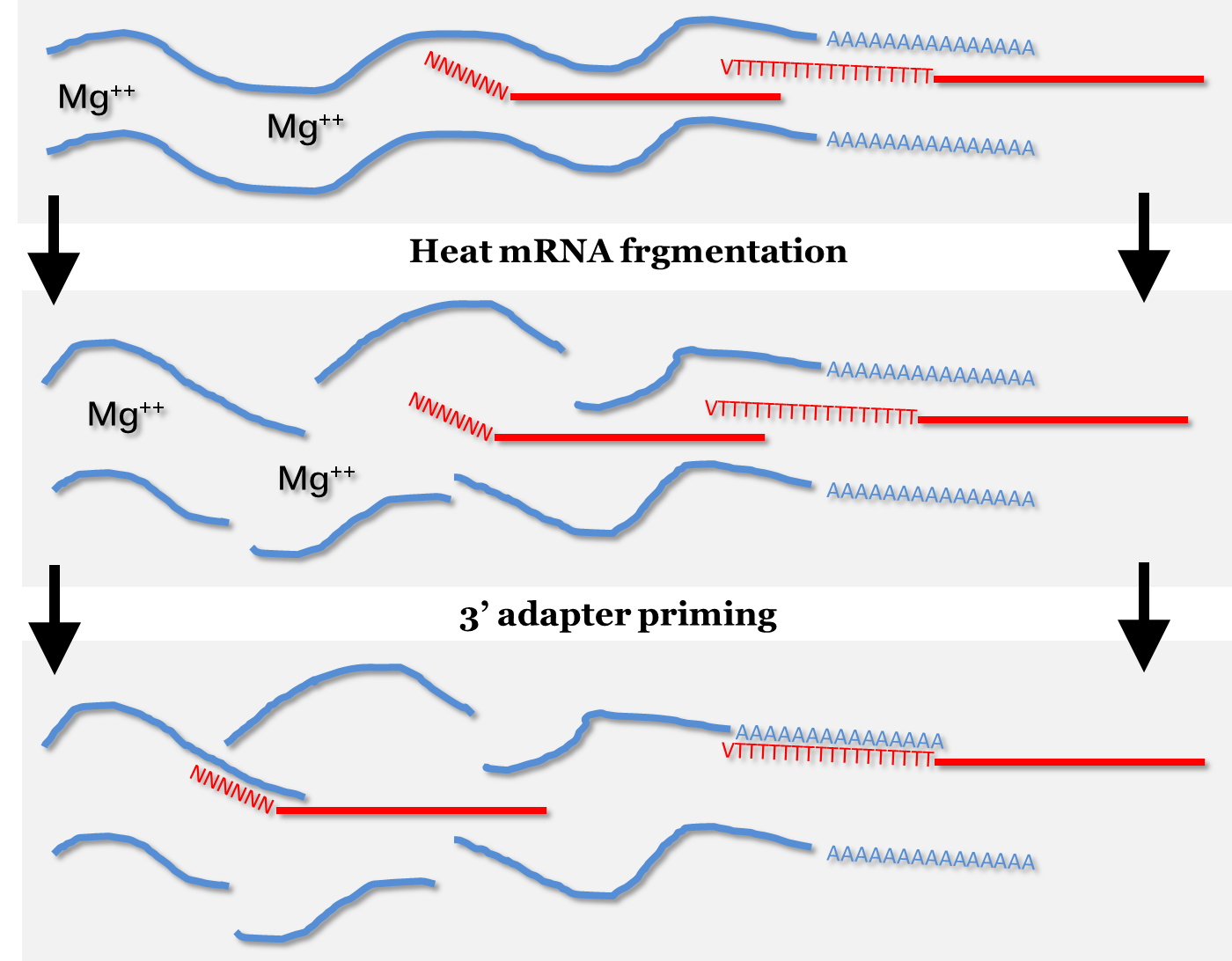


**Procedure:**

● Place 1.5 μl 5X Thermo Scientific RT buffer and 1 μl 3-prime priming adapter into fresh tube for each sample. (It is convenient to make up a large volume of 1.5:1 FS-buffer + Priming-Oligo so that 2.5 μl can be directly dispensed for each RNA or DNA sample.)

*If using **Superscript II** or **Superscript III** instead of Thermo RT, use 2 ul of buffer and adjust RNA volume accordingly to assure the correct magnesium concentration for RNA fragmentation.

DGE: mRNA polyA-adapter priming: 1 μl 2 μM oligo L-3ILL-20TV.2

SHO: mRNA random octamer-adapter priming: 1 μl 5 μM oligo L-3ILL-N8.2

CNV: mRNA for non strand-specific: .5 μl Invitrogen random primers

● Transfer 7.5 μl of RNA or fractured DNA (8 ul of RNA or DNA when using random primers) to each tube and mix well by pipetting. Place remaining RNA or fractured DNA in -20 or -80 for troubleshooting or future library preps. (make up the difference in volume with sample or H2O if using Invitrogen random primers. They are expensive and .5 μl works as well as 1 μl)

● Spin down samples to ensure all of sample is at the bottom of the tube.

● Place in thermocycler for fragmentation and 1^st^ strand priming.

Keep fragmented **polyT** primed mRNA at room temperature and set up the reverse transcription reaction at room temperature. Hold thermocycler block at 25C prior to transferring samples.

Keep fragmented **random primer** and **Random Octomer adapter**-primed mRNA on ice and set up reverse transcription reaction on ice then transfer to room temperature

Fragmentation/Priming program:

**DGE polyT**-adapter primed:

(25°C 1 second, 94°C 1.5 min, 30°C 1 min, 20°C 4 min, 20°C hold)

**SHO random Octomer**-adapter **and random primer** primed: (25°C 1 second, 94°C 1.5 min, 4°C 5 min, 4°C hold)


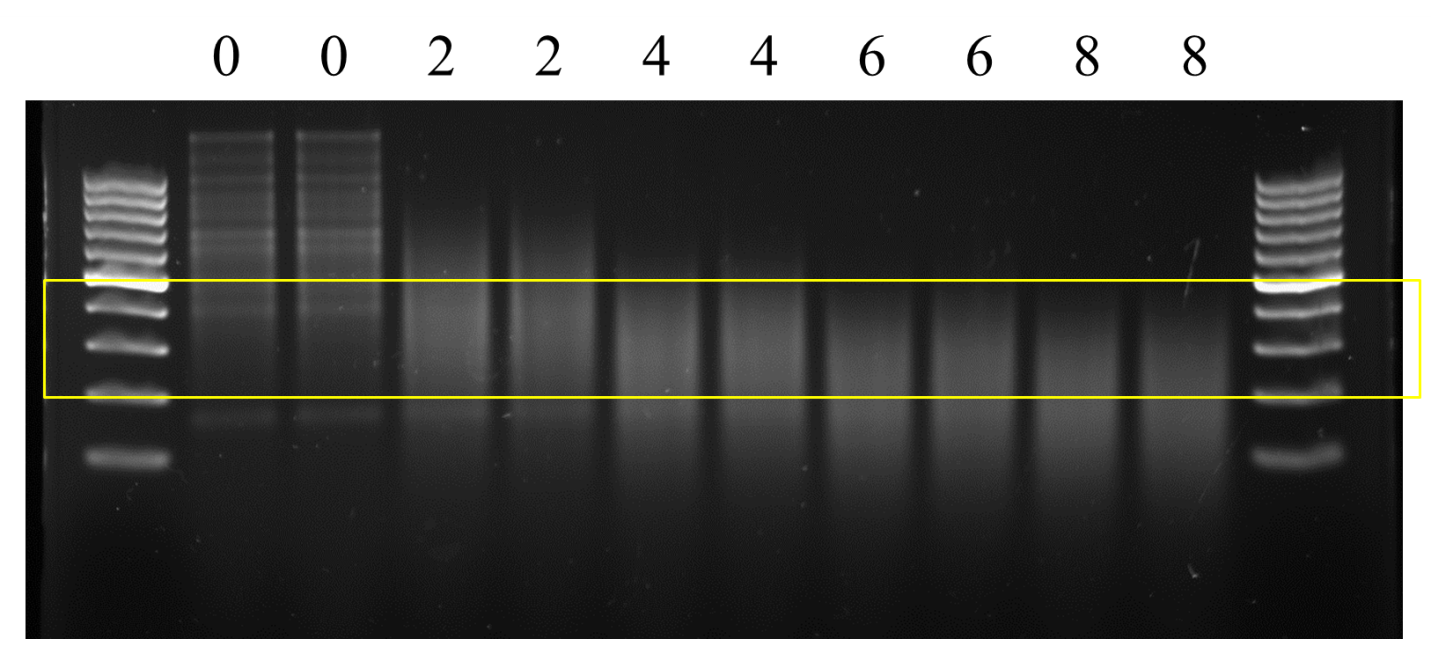


Fragmentation time (minutes) of isolated RNA at 94C.

Oligonucleoties used in this section:

L-3ILL-20TV.2 GTGACTGGAGTTCAGACGTGTGCTCTTCCGATCTTTTTTTTTTTTTTTTTTTTV

L-3ILL-N8.2 GTGACTGGAGTTCAGACGTGTGCTCTTCCGATCTNNNNNNNN

**3: cDNA Synthesis**

**cDNA synthesis is the same for strand specific and non-strand specific libraries although the illustration only shows strand-specific examples.**


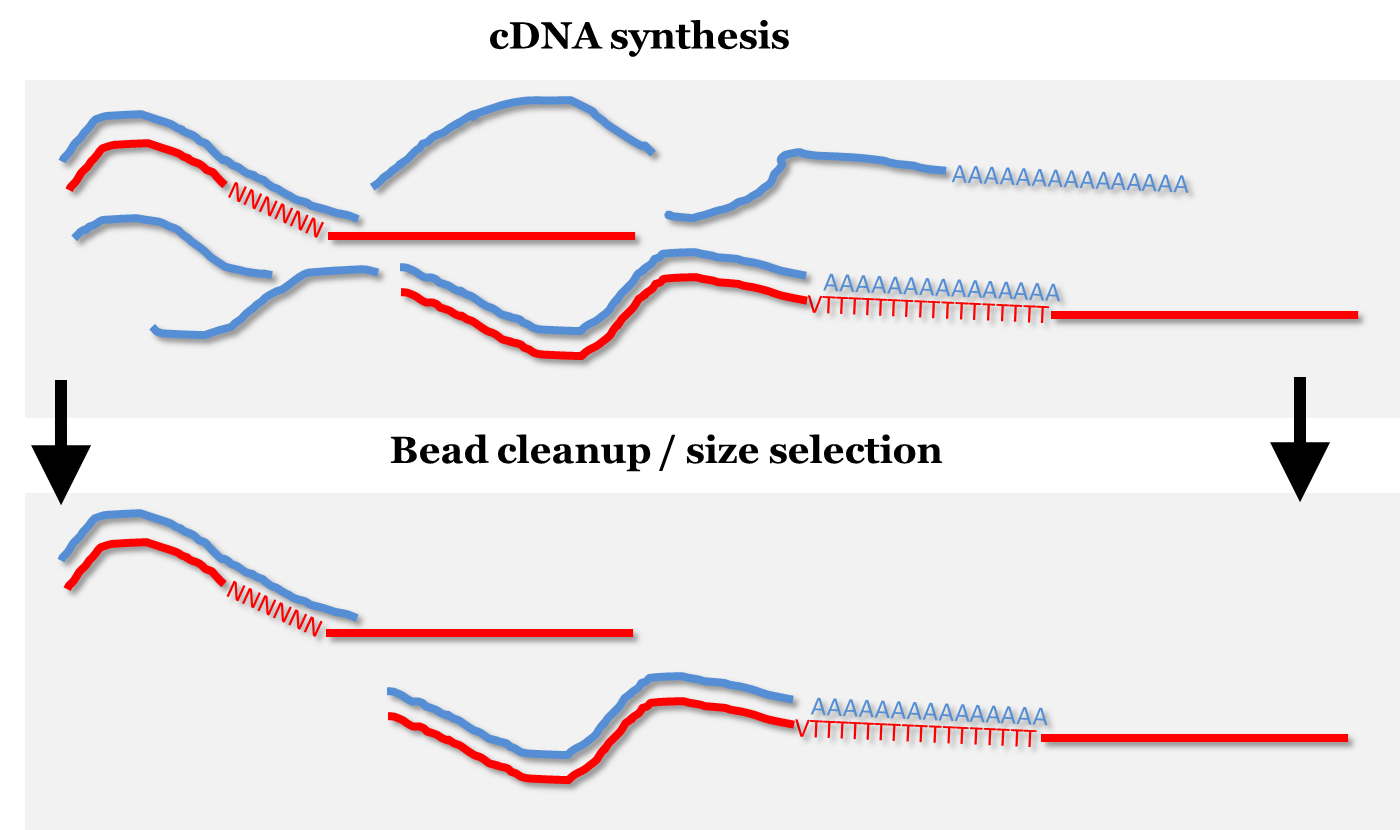


Solutions required:

● Ampure XP Bead Resuspension Buffer (**ABR**) (Store at room temperature, remake after a few months because it goes bad and will not bind DNA to the beads any longer)

Solution components Final concentration

PEG 8000 15%

NaCl 2.5 M

Procedure:

Prepare 1^st^ strand master mix.

Reagent vol/rxn | | |

5X Thermo Scientific RT buffer 1.5 μl | | |

.1M DTT 1.5 μl | | |

H2O 1 μl | | |

25mM dNTPs .5 μl | | |

RevertAid RT enzyme .5 μl | | |

*RNAse OUT 0 μl

*Do not use RNAse OUT unless necessary. Expensive and actually reduces library output

*If using **Superscript**, adjust buffer and water volumes to 1 μl and 1.5 μl respectively.

Add 5 μl of mix to each fragmented RNA sample and mix well.

Total rxn volume 15 μl

● Incubate in thermocycler for reverse trascription step with the following program.

(25°C 10 min, 42°C 50 min, 50°C 10 min, 70°C 10 min, 4°C hold)

**If cDNA is for Conventional (non-strand specific) libraries precede directly to module 6.1 without doing the wash steps below.**

● Add 5 μl of 50 mM EDTA pH 8.0 and 30 μl Ampure beads to each sample. (1.5X Ampure/sample volume).

● Mix well and let stand 5 min at room temperature

● Place on magnetic tray and remove supernatant.

● Wash 2X with 300 μl of 80% EtOH without resuspending pellet. Allow pellets to dry.

*Avoid significantly over drying beads or beads may clump and be difficult to homogenize.*

*The protocol can be paused at any point where the beads are in 80% EtOH at the second wash by storing the strip tubes/plates at -20C, even for an extended period of many weeks if necessary.*

**4: Double stranded adapter annealing**

Both the strand specific and the non-strand specific libraries use double stranded adapters at some point in their protocol. Non-strand specific libraries use a “Y” shaped adapter which is ligated to both ends of a double stranded DNA molecule, whereas the strand specific libraries use a single stranded adapter to prime cDNA synthesis and a double strand adapter to capture the cDNA in a separate step. The adapters should be prepared in advance and can be stored at -20 C indefinitely.

**Strand specific 5’ adapter annealing**

**Procedure:**

● hydrate the main stocks of sense and antisense 5-prime adapter oligos when they arrive to a concentration of 100 μM.

● prepare adapters by adding 80 μl of 100 μM 5pSense8n oligo and 80 μl of 100 μM 5pAnti oligo. Add 640 μl of H2O, vortex and spin down. Aliquot 100 μl in 8 strip flip-cap strip tubes and spin the strip so that all droplets are collected and run the following annealing program.

(94 C, 1min (94 C, 10 sec) X 60 cycles -1 C/cycle, 20 C 1 min, 4 C hold)

The final concentration should be 10 μM of the double stranded 5’prime adapter

It is a good idea to use different colors of flip-cap 8 strip tubes for the different types of adapters to avoid confusion.

OLIGO SEQUENCES:

5pSense8n CCTACACGACGCTCTTCCGATCTnnnnnnnn

5pAnti AGATCGGAAGAGCGTCGTGTAGG

**Non-strand specific “Y” adapter annealing**

**Procedure:**

● hydrate the main stocks of sense and antisense 5-prime adapter oligos when they arrive to a concentration of 100 μM.

● prepare adapters by adding 8 μl of 100 μM PE1-lig oligo and 8 μl of 100 μM 5’ phosphorolated ILL-lig oligo. Add 784 μl of H2O, vortex and spin down. Aliquot 100 μl in 8 strip flip-cap strip tubes and spin the strip so that all droplets are collected and run the following annealing program.

(94 C, 1min (94 C, 10 sec) X 60 cycles -1 C/cycle, 20 C 1 min, 4 C hold)

The final concentration should be 1 μM of the Y shaped ligation adapter

It is a good idea to use different colors of flip-cap 8 strip tubes for the different types of adapters to avoid confusion.

OLIGO SEQUENCES:

PE1-lig CACTCTTTCCCTACACGACGCTCTTCCGATCT      
ILL-lig P-GATCGGAAGAGCACACGTCTGAACTCCAGTCAC

**5: 5-prime adapter sequence addition (on bead)**


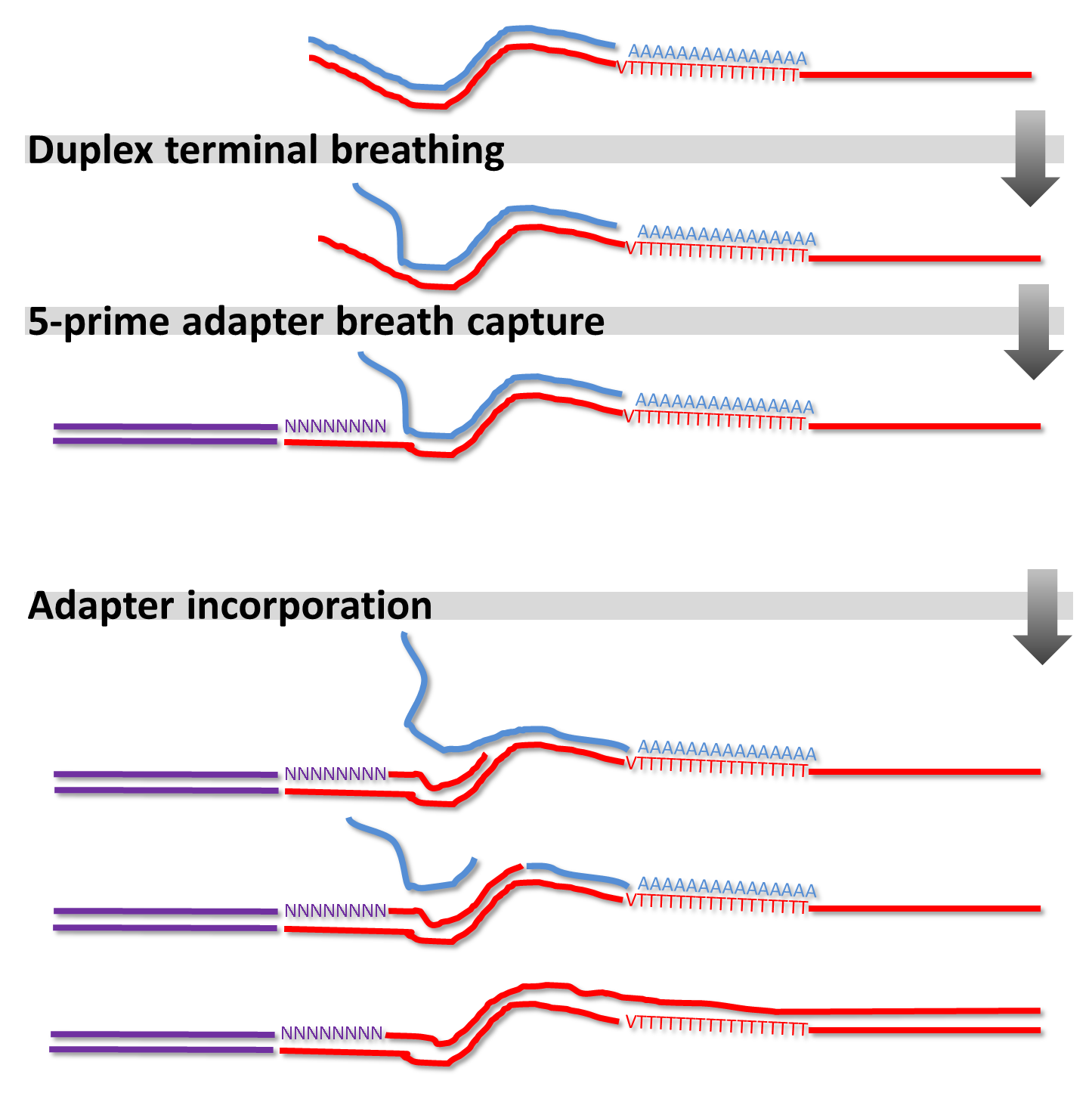


5-prime adapter annealing

The 5’ adapter is partially double stranded so that the adapter sequence is shielded from interaction with the cDNA to prevent sequence bias in cDNA adapter addition. Prepare annealed double-stranded 5’ adapter before beginning this step.

**Procedure:**

Make sure the 5-prime adapters are mixed and annealed before starting.

Prepare adapter extension master mix in advance during cDNA synthesis but add enzyme immediately prior to use. Keep reaction mix and cDNA samples at room temperature.

● Thaw 10 μM working stocks of 5-prime adapter and then allow them to warm to room temperature before use. Make sure to shake tubes and briefly spin down to homogenize melted ice crystals and condensation.

● Directly to the dry bead pellet add 4 μl room temperature 10 μM 5-prime double stranded adapter oligo. It is not necessary to resuspend the beads at this point.

10 mM 5-prime adapter 4 μl

Reaction mix vol/rxn | | |

H2O 3.5 μl | | |

10X PolI buffer 1 μl | | |

250 mM MgCl2 1 μl | | |

25 mM dNTPs .25 μl | | |

DNA Pol I .25 μl | | |

● Add 6 μl reaction cocktail to each sample and mix well by pipetting resuspending the beads.

Total Rxn vol. 10 μl

● Incubate at room temperature for 15 minutes. (Approximately 25 C)

● Add 10 μl 50 mM EDTA pH 8.0 and 30 μl ABR (1.5X Ampure/sample volume) to each sample.

● Mix well to resuspend Ampure beads and let stand 5 min at room temperature.

● Place on magnetic tray and remove supernatant

● Wash 2X with 300 μl of 80% EtOH without re-suspending pellet. Allow pellet to dry.

● Resuspend pellet in 20-22 μl 10mM Tris pH 8.0

Small samples: Resuspend pellet in 12 μl 10mM Tris pH 8.0

Optional: 30 μl of ABR can be added and Ampure purification should be repeated prior to enrichment if small amplicon sizes or adapter contamination are a problem. (This is not for small samples.)

● Transfer supernatant to fresh tubes.

● Add 200-300 μl mili-Q H20 to ampure beads and re-suspend. Keep on ice for post-enrichment cleanup.

**6.1: Non-strand-specific RNAseq libraries**

This module is for preparation of random primer primed cDNA made from fragmented mRNA into non-strand specific libraries. The various enzymatic steps can occur in the same buffer conditions and do not mutually interfere.

**A: Second strand synthesis, end preparation and A-tailing**

● Make second strand master mix vol/rxn | | |

H2O 1.5 μl | | |

25mM dNTPs .4 μl | | |

DNA PolI 1 μl | | |

RNAseH .1 μl | | |

End Repair enzyme mix .4 μl | | |

Taq poymerase .2 μl | | |

End repair buffer 1.4 μl | | |

● Add 5 μl of mix to each sample on ice. Mix and spin down strip.

● Place in thermocycler and run following program: (16C 20m, 20C 20m, 72C 20m, 4C Hold)

● Add 30 μl Ampure XP beads, mix and let stand at RT for 5 minutes.

● Magnetize and wash 2x with 300 ul 80% EtOH.

● Leave strip on magnetic rack and allow beads to dry

**B: Adapter ligation**

● Add 3 μl of annealed 1 μM universal adapters to each dry bead pellet.

● Add 7 μl of the following mix to each sample and mix by pipetting up and down making sure to resuspend Ampure beads.

● Master ligation mix vol/rxn | | |

H2O 1.75 μl | | |

2X rapid T4 ligase buffer 5.0 μl | | |

T4 DNA ligase .25 μl | | |

● Place strip lid on to avoid evaporation and allow to stand at Room Temperature for 15 minutes.

● Add 10 μl of 50 mM EDTA to each sample

● Add 25 μl of ABR to each sample and mix by pipetting up and down.

● Allow to stand 5 min at RT

● Magnetize and wash 2x with 300 μl 80% EtOH.

● Leave strip on magnetic rack and allow beads to dry

● When beads are dry add 20-22 μl 10mM Tris to each sample and re-suspend beads.

● Magnetize and transfer liquid to new strip tubes.

● Proceed to enrichment.

**6.2: double-stranded DNA library prep**

This module is for any double-stranded DNA input material already in the size range of library input material such as digested genomic DNA or amplified CHIP material.

**A: End preparation and A-tailing**

Input sample volume = 15 μl

● Make second strand master mix vol/rxn | | |

H2O 2.7 μl | | |

25mM dNTPs .4 μl | | |

End Repair enzyme mix .4 μl | | |

Taq poymerase .2 μl | | |

End repair buffer 1.5 μl | | |

● Add 5 μl of mix to each sample on ice. Mix and spin down strip.

● Place in thermocycler and run following program: (16C 20m, 20C 20m, 72C 20m, 4C Hold)

● Add 30 μl Ampure XP beads, mix and let stand at RT for 5 minutes.

● Magnetize and wash 2x with 300 ul 80% EtOH.

● Leave strip on magnetic rack and allow beads to dry

**B: Adapter ligation**

● Add 3 μl of annealed 1 μM universal adapters to each dry bead pellet.

● Add 7 μl of the following mix to each sample and mix by pipetting up and down making sure to resuspend Ampure beads.

● Master ligation mix vol/rxn | | |

H2O 1.75 μl | | |

2X rapid T4 ligase buffer 5.0 μl | | |

T4 DNA ligase .25 μl | | |

● Place strip lid on to avoid evaporation and allow to stand at Room Temperature for 15 minutes.

● Add 10 μl of 50 mM EDTA to each sample

● Add 25 μl of ABR to each sample and mix by pipetting up and down.

● Allow to stand 5 min at RT

● Magnetize and wash 2x with 300 μl 80% EtOH.

● Leave strip on magnetic rack and allow beads to dry

● When beads are dry add 20-22 μl 10 mM Tris to each sample and re-suspend beads.

● Proceed to enrichment.

**6.3: Single-stranded DNA input material**

This module is for use of single stranded DNA as an input material for library prep.

**A: DNA denaturation and priming**

● Second strand synthesis master mix vol/rxn | | |

DNA sample + H2O 13.9 μl | | |

10X PolI buffer 0.6 μl | | |

Random primers (Invitrogen) 0.5 μl | | |

● Mix and spin down strip

● Place in thermocycler and run following program: (94C 1m30s, 4C 5m, 4C Hold)

● Place strip on ice for second strand setup.

**B: Second strand synthesis, end preparation and A-tailing**

● Make second strand master mix vol/rxn | | |

H2O 1.6 μl | | |

25mM dNTPs .4 μl | | |

DNA PolI 1 μl | | |

End Repair enzyme mix .4 μl | | |

Taq poymerase .2 μl | | |

End repair buffer 1.4 μl | | |

● Add 5 μl of mix to each sample on ice. Mix and spin down strip.

● Place in thermocycler and run following program: (16C 20m, 20C 20m, 72C 20m, 4C Hold)

● Add 30 μl Ampure XP beads, mix and let stand at RT for 5 minutes.

● Magnetize and wash 2x with 300 μl 80% EtOH.

● Leave strip on magnetic rack and allow beads to dry

**C: Adapter ligation**

● Add 3 μl of annealed 1 μM universal adapters to each dry bead pellet.

● Add 7 μl of the following mix to each sample and mix by pipetting up and down making sure to resuspend Ampure beads.

● Master ligation mix vol/rxn | | |

H2O 1.75 μl | | |

2X rapid T4 ligase buffer 5.0 μl | | |

T4 DNA ligase .25 μl | | |

● Place strip lid on to avoid evaporation and allow to stand at Room Temperature for 15 minutes.

● Add 10 μl of 50 mM EDTA to each sample

● Add 25 μl of ABR to each sample and mix by pipetting up and down.

● Allow to stand 5 min at RT

● Magnetize and wash 2x with 300 μl 80% EtOH.

● Leave strip on magnetic rack and allow beads to dry

● When beads are dry add 20-22 μl 10 mM Tris to each sample and re-suspend beads.

● Proceed to enrichment.

**10.1: Enrichment and adapter extension**


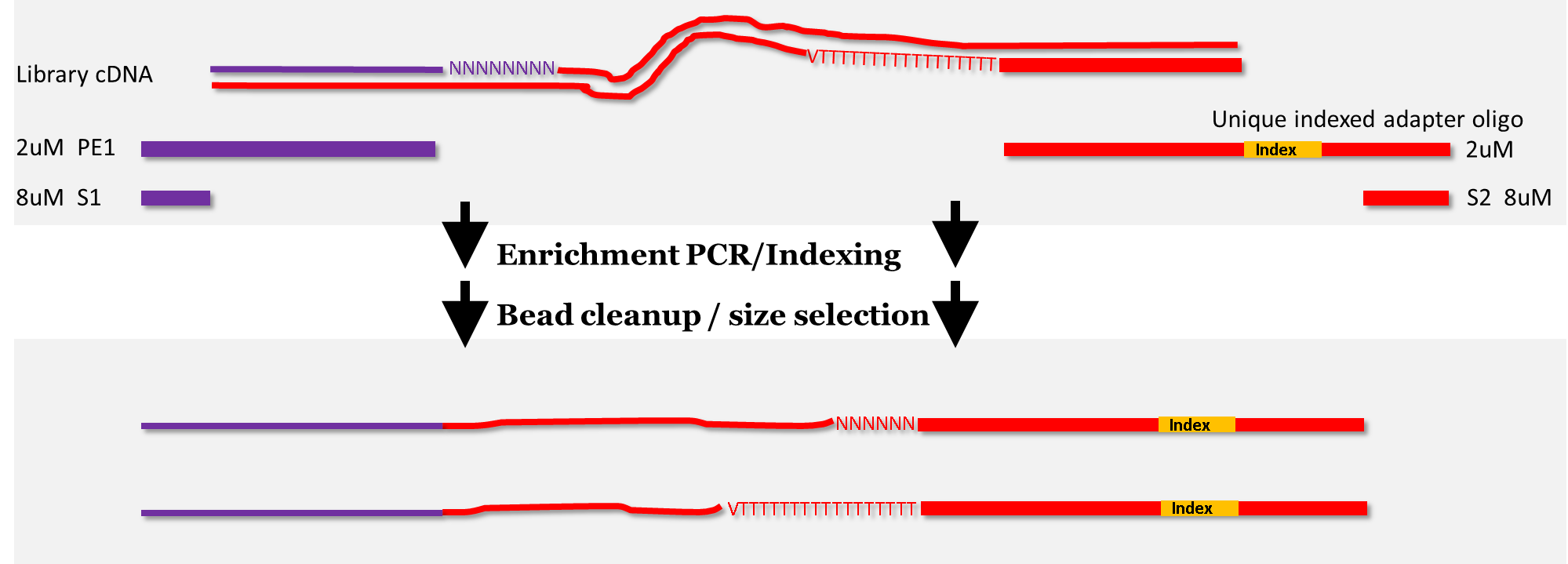


The enrichment PCR uses four primers concurrently, two long primers to complete the adapter sequence at low concentration, and two short primers consisting of the terminal most sequence of the adapters at higher concentration. This is to selectively amplify molecules with complete adapter sequences, since topo cloning and sanger sequencing of library molecules showed a high percentage of individual library molecules contained only partial adapter sequences presumably due to “broken” oligos. Consequently, amplification of adapter contamination was significantly reduced as for reasons unknown.

Set up enrichment at room temperature. Hot start is not necessary.

● Make enrichment master mix vol/rxn | | |

5X Phusion HF Buffer 4 μl | | |

H2O 2.6 μl | | |

2 μM PE1 primer 1 μl | | |

8 μM each EnrichS1 + S2 primers 1 μl | | |

25mM dNTPs .2 μl | | |

Phusion Polymerase .2 μl | | |

● Add 9 μl of master mix to each well 9 μl

● Transfer 1 μl of appropriate unique indexed enrichment oligo to each well

*2 μM ILL-INDEX primer 1 μl

● Transfer 10 μl of adapterized cDNA to each well 10 μl

Mix well by pipetting up and down

total 20 μl Rxn

● Without changing tips from previous step, transfer 10 ul of enrichment PCR mix to second 8 strip PCR tubes [**Optional but HIGHLY recommended**, see below]

*ILL-BC is the index barcoding adapter primer

**! Hedge your bets: Particularly with small samples or any time you don’t have a reasonable idea how many amplification cycles will provide sufficient amplification without over-amplifying, transfer 10 μl of the pre-amplification reaction volume and freeze at -20 C, then run the remainder of the reaction volume and run 2 ul on an agarose gel. This will allow you to adjust the number of cycles for the remainder of the reaction volume. The remaining 8 ul of enriched libraries should provide enough sample for the final cleanup and pooling.**

● Incubate in thermocycler with following program

98 C 30 seconds, (98 C 10 seconds, 65 C 30 seconds, 72 C 30 seconds) *N cycles, 72 C 5 min, 10 C hold.

(Depending on your particular samples the number of cycles may be less (or more) than the recommended number, but these are a good starting point for new materials)

*For **polyT**-adapter primed libraries use 11+- cycles

*For **Hex**-adapter primed libraries use 14+- cycles

Small samples: Start out by using ~3 more cycles than you otherwise would.

● Run 2 μl on 1% Agarose gel for 20 minutes at 100 Volts to check results. If desired amplification is obtained proceed to cleanup.

● If desired amplification is not obtained for all samples, use remaining adapter-added cDNA for enrichment with increased number of cycles.


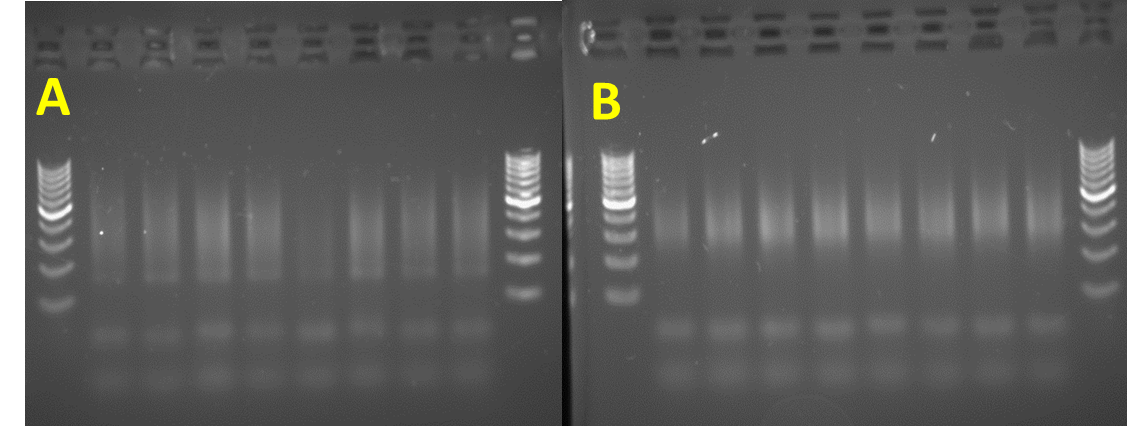


2 ul of enrichment reaction loaded on to 1% SB gel run 20 minutes at 100 volts. Ladder used is 1 ul of Thermo Scientific O’GeneRuler 100bp DNA ladder.

1. 3’DGE strand-specific RNA-seq libraries run at 11 cycles.
2. Non-strand specific RNA-seq libraries run at 9 cycles.

The strand specific RNA-seq libraries produce a broader size distribution than the non-strand-specific libraries. The smaller library molecules are removed in the subsequent wash steps.

**8: Final library cleanup**

**Using fresh Ampure beads**

● Add 1.5 volumes (i.e. 12 μl for 8 ul of enrichment product) resuspended Ampure beads to sample, mix well and let sit at room temperature for 5 minutes. (If large amounts of adapter contamination is evident use Ampure bead ratio of 1.2:1, bring sample to 10ul with 10 mM tris and add 12 μl of beads)

● Place on magnetic tray and remove supernatant

● Wash 2X with 300 μl of 80% EtOH without resuspending pellet. Allow pellet to dry.

● Re-suspend pellet in 10 μL 10mM Tris pH 8.0

2 μl can be run on a gel to make sure of sufficient recovery and complete lack of adapter contamination.

● Proceed to quantification and pooling. Quantification can be done by any number of methods but is done in order to pool equal amounts of each library for sequencing. An additional was of the pooled libraries is generally done to ensure removal of any remaining adapter contaminants.
